# Predator scent stress reduces oxycodone self-administration and the nucleus accumbens dopamine response to oxycodone in female rats

**DOI:** 10.1016/j.addicn.2025.100210

**Published:** 2025-06

**Authors:** Courtney S. Wilkinson, Siya Bhutani, Wonn Pyon, Marek Schwendt, Lori A. Knackstedt

**Affiliations:** a Psychology Department, 945 Center Drive, University of Florida, Gainesville, FL, USA; b Center for Addiction Research and Education, 1395 Center Dr, Suite D2-013, University of Florida, Gainesville, FL, USA; c Department of Neuroscience, 1149 Newell Drive, University of Florida, Gainesville, FL, USA

**Keywords:** Anhedonia, Substance use disorder, Addiction, Relapse

## Abstract

Post-traumatic stress disorder (PTSD) develops in a sub-population of people exposed to trauma and is highly comorbid with opioid use disorder (OUD). The neurobiology of comorbid PTSD+OUD, especially with respect to sex differences, is poorly understood. Here we investigated the sex-specific effects of predator scent stress on intravenous (IV) oxycodone self-administration (SA), cue-primed reinstatement, and pre- and post-stress corticosterone (CORT) concentrations. Upon detecting effects of stress on oxycodone IVSA, the nucleus accumbens (NA) dopamine response to oxycodone was assessed. Male and female rats received a single 10-minute exposure to the predator scent TMT or the control condition. One week later, rats were tested for anxiety-like behavior on the elevated plus maze and underwent oxycodone IVSA, instrumental extinction, and a cue-primed reinstatement test. In a separate cohort of only female rats, the NA core dopamine response to IV oxycodone (0, 0.25, 0.5 mg/kg) was assessed using fiber photometry. TMT increased anxiety-like behavior one week later in male and female rats. Baseline CORT was negatively correlated with anxiety-like behavior in males. TMT exposure reduced oxycodone intake exclusively in females, with no effects on instrumental extinction or cue-primed reinstatement in either sex. Female rats demonstrated a dose-dependent increase in NA core dopamine signal following oxycodone administration that was blunted by a history of TMT exposure. These results indicate that TMT exposure diminishes the NA core dopamine response to oxycodone in females, reducing oxycodone IVSA. These results are consistent with stress-induced reward insensitivity that has been observed following stress in both rodents and humans.

## 1. Introduction

The lifetime prevalence of developing PTSD after trauma exposure is approximately 8–30 % with a higher prevalence in women than men [1–4]. Those diagnosed with PTSD are two to six times more likely to develop a substance use disorder (SUD) than those without, with individuals with opioid use disorder (OUD) displaying a particular risk for comorbid PTSD [2,5–8]. Individuals with comorbid PTSD+OUD show worse treatment outcomes than those with either disorder alone [9,10]. Despite the high prevalence of comorbid PTSD+OUD, there is limited research on the overlapping neurobiology of these disorders.

Animal models of PTSD+OUD can be useful for the identification of neurobiology underlying both disorders in the context of comorbidity. The predator scent stress (PSS) model has utility for these investigations, as a single exposure to PSS produces long-lasting effects on fear and anxiety-like behaviors [11,12]. Additionally, weeks after PSS exposure, increased drug-seeking has been observed across several classes of drugs. Male rats exposed to PSS later increase alcohol self-administration [13]. Rats with greater fear/anxiety-like behavior after PSS demonstrate greater alcohol consumption [14] and show more persistent responding for alcohol [15]. The same pattern is true for cocaine self-administration [16,17]. Additionally, male rats with greater anxiety-like behavior show instrumental extinction deficits and greater cue-primed reinstatement of cocaine-seeking [18]. Increased cue-primed methamphetamine-seeking is also observed in male rats repeatedly exposed to PSS [19].

Individuals with PTSD report a higher rate of prescription opioid misuse than those without PTSD [9,20–23]. The effects of PSS on opioid self-administration have yet to be assessed, particularly in female rodents. Therefore, the present experiment investigates the effects of a single, brief exposure to the fox scent 2,4,5-trimethyl-3-thiazoline (TMT) on oxycodone intake and seeking in male and female rats. Unlike our prior work, which used a double-median split analysis of anxiety-like behavior to identify subpopulations of PSS-susceptible and -resilient rats [18,24,25], here we sought to identify relationships between anxiety-like behavior and oxycodone self-administration that may exist on a continuum. Our hypothesis, based on prior work with alcohol, methamphetamine, and cocaine, was that TMT exposure would increase oxycodone self-administration, impair instrumental extinction, and increase oxycodone-seeking during a cue-primed reinstatement test in both sexes. However, our data did not support these hypotheses, revealing that female TMT-exposed rats self-administered less oxycodone than control rats. As we previously found that a single exposure to TMT alters dopamine turnover in the female nucleus accumbens (NA) core [24], and dopamine signaling in the NA core is necessary for reinforcement, we next assessed the effects of TMT exposure on the NA core dopamine response to oxycodone in female rats.

In humans, a reduced cortisol response in the first 15 h following a traumatic event increases the risk of a later PTSD diagnosis [26–28]. There is preclinical evidence that blunted basal CORT pulse amplitude increases stress-susceptibility. Further, Lewis rats, which have the greatest PSS-induced anxiety-like responses compared to Sprague Dawley and Fischer rats, have a blunted corticosterone (CORT) response to PSS compared to the other strains [29]. When treated with exogenous CORT administration an hour before PSS or an hour after PSS, Lewis rats demonstrate resilience to anxiety-like behavior one week later [29,30]. Thus, a secondary goal of the present work was to test the hypothesis that rats with lower basal CORT and a blunted CORT response to PSS would demonstrate greater anxiety-like behavior and be more susceptible to oxycodone-seeking.

## 2. Methods

### 2.1. Subjects

One-hundred and twenty-two adult Sprague-Dawley rats (47 male, 75 female; Charles River Laboratories, Raleigh, North Carolina) were individually housed on a 12-hr reversed light cycle (lights off at 7 am) in a humidity and temperature-controlled vivarium. Water was provided ad libitum. Rats were provided 20 g of chow per day, resulting in a modest increase in body weight over the course of the study (male: 4.48 % increase; female: 4.44 % increase). TMT/Control exposure, anxiety assessments, and blood collection for CORT analysis were conducted during the first 5 h of the dark cycle due to circadian rhythms of HPA axis activity, and to be consistent with our prior work using this model [18,25,31,32]. Self-administration sessions were conducted during the dark phase. Fiber photometry sessions were counterbalanced such that half of each group was tested between 0800–1200 and the other half from 1200–1600 to control for the known effect of circadian rhythm on NA dopamine dynamics [33]. All procedures were approved by the Institutional Animal Care and Use Committee at the University of Florida.

### 2.2. Drugs and chemicals

Oxycodone HCl (Sigma Aldrich, St. Louis, MO) was dissolved in 0.9 % sterile physiological saline, which was used as the vehicle (VEH). During oxycodone intravenous self-administration (IVSA), different oxycodone concentrations were used for male (0.4 mg/mL) and female (0.32 mg/mL) rats due to weight differences between sexes so that all rats received 0.1 mg/kg/infusion in a similar volume of liquid. A 3 mg/mL concentration of oxycodone was passively administered at a 0.25 or 0.5 mg/kg (IV) dose during photometry sessions. 2,4,5-trimethyl-3-thiazoline (TMT; 97 % purity) was obtained from BioSRQ (Sarasota, FL).

### 2.3. Predator scent stress exposure

Rats were subjected to a single, 10-minute exposure to 3 μL of TMT or the unscented control condition. Exposures occurred in a plexiglass cylindrical container (Bio Bubble Pets, Boca Raton, FL; 40 cm diameter x 35 cm height). Below a steel mesh floor was a cup where TMT-saturated or unscented filter paper was placed immediately prior to each exposure. Separate chambers were used for the control and TMT conditions. Exposure sessions were recorded, and freezing behavior was scored by reviewers that were blind to experimental conditions. Freezing was defined as a lack of movement, with the exception of respiration. Test chambers were cleaned with 70 % ethanol between sessions.

### 2.4. Elevated plus maze

Rats were tested for anxiety-like behavior using the elevated plus maze (EPM) one week after TMT exposure. The EPM (Med Associates, St. Albans, VT) consisted of two opposing open arms and two opposing closed arms (L x W x H: 51×10×40.5 cm) and a center zone (L x W: 10×10 cm) room illuminated to 50 lx. Rats were placed in the center zone and allowed to freely explore for 5-minutes. The amount of time spent in open and closed arms and the number of closed and open arm entries were recorded using Ethovision XT 14 software (Noldus Information Technology, Leesburg, VA). Lower amounts of time spent in and lower number of entries to the open arms were interpreted as indicators of greater anxiety-like behavior [34]. Test chambers were cleaned with 70 % ethanol between sessions.

### 2.5. Blood collection and CORT analysis

Blood was collected into pretreated K3 EDTA (12 mg) BD Vacutainers (BD, Franklin Lakes, NJ) and centrifuged at 1600 g for 15 min. Plasma was extracted and frozen at − 80 °C for later analysis of CORT via ELISA (Enzo Life Sciences, Farmingdale, NY) using the manufacturer’s protocol (which included steroid dissociation reagent incubation).

### 2.6. Surgery

Rats were anesthetized with ketamine (males: 87.5 mg/kg, IP; females: 60 mg/kg, IP) and xylazine (5 mg/kg, IP) and surgically implanted with indwelling jugular catheters for IVSA or a non-contingent IV infusion during fiber photometry sessions. Silastic catheters (SILASTIC, ID 0.51 mm, OD 0.94 mm, Dow Corning, Midland, MI) were implanted into the jugular vein and secured with suture thread. Catheter tubing was run subcutaneously through a small incision in the back, connected to a cannula (Plastics One, Roanoke, VA), and secured to a harness (Instech, Plymouth Meeting, PA). Rats were administered the antibiotic cefazolin (100 mg/mL, IV) and the analgesic carprofen (1 mg/kg, SC) on the day of surgery and for three days post-operatively. Catheters were flushed daily with heparinized saline (100 mg/mL; 0.1 mL) to maintain patency. Catheter patency was verified weekly with methohexital sodium (10 mg/mL, IV; Eli Lilly, Indianapolis, IN).

Rats in Experiment 2 received AAV infusion and optic fiber implantation immediately following catheter implantation. For AAV infusion, a microinfusion pump was used to deliver 1 μL of the dopamine sensor GRAB_DA2m_ (AAV9-hSyn-GRAB_DA2m; 1 × 10^12^; Addgene, Watertown, MA [35]) into the NA core (AP +1.5, ML ± 1.5, DV − 5.8) of one hemisphere (in a counterbalanced manner in each group). The flow rate was 0.14 μl/minute and the microinjector was kept in place for 10 min after the infusion. A 400 μM optical fiber (Doric Lenses, Quebec City, Quebec) was then implanted using the same coordinates as the viral infusion. Optical fibers were secured to the skull with dental cement and three stainless steel surgical screws.

### 2.7. Oxycodone IVSA

Rats underwent 3h oxycodone IVSA sessions for 12 days in standard operant chambers (30×24×30 cm; Med Associates, St. Albans, VT) within sound attenuating cubicles with ventilation. Chambers were equipped with a house light, a tone generator, and a stimulus light above each of the two levers. The house light was illuminated for the duration of each session. Each drug delivery resulted in a 2.5 s IV oxycodone infusion (0.1 mg/kg/infusion), akin to the dose used in Altshuler et al. [36], and a 5-second presentation of a white stimulus light and a 78 dB, 4.5 kHz tone, followed by a 20-second time out period where lever presses were documented but had no programmed consequences. All responses were recorded using MedPC-V software. Rats self-administered oxycodone on a fixed ratio 1 (FR1) schedule of reinforcement for six days, followed by six days on an FR3 schedule of reinforcement.

After 12 days of IVSA, rats that met SA criteria (earning ≥ 10 infusions/day for 9 out of 12 sessions) underwent daily instrumental extinction sessions (2 hr/day) where presses on both levers were recorded but did not result in programmed consequences. Extinction sessions continued for a minimum of 9 days. Rats that met extinction criteria (<20 % of the average active lever presses on the last day of self-administration) then underwent a 60-minute cue-primed reinstatement test where cues were presented in response to active lever presses on an FR1 schedule of reinforcement without drug delivery.

### 2.8. Vaginal lavage

Due to the effect of estrous cycle on the mesolimbic dopamine system of female rats, estrous cycle was tracked for four days prior to fiber photometry recordings using vaginal lavage [37–39]. Vaginal lavage was performed approximately 30 min prior to sessions. Ultrapure water was lightly washed along the vaginal opening, collected, and placed on a slide for later phase determination using a 10x light microscope. After drying, lavage samples were stained using crystal violet and phase of estrous was determined by cell type according to McLean et al. [40]. Proestrus was determined by a majority of nucleated epithelial cells with early stages of cornification. Estrus was determined by large cornified cells in dense clusters. Metestrus was determined by the presence of a majority of leukocytes. Diestrus was determined when leukocytes and nucleated epithelial cells were observed.

### 2.9. Fiber photometry recording, preprocessing, and analysis

In Experiment 2, rats underwent fiber photometry sessions. Photometry signals were recorded through an RZ10 Lux Integrated Processor (Tucker Davis Technologies, Alachua, FL) equipped with 465 nm and 415 nm LEDs to record dopamine-dependent and isosbestic control signals, respectively. Signals were sampled at 1017 Hz. Dopamine signals were analyzed using custom MATLAB (Mathworks, MA) scripts adapted from Dr. David Root (University of Colorado, Boulder) and inspired by the workflow developed by Barker et al. [41]. Isosbestic (415 nm) and dopamine-dependent (465 nm) signals were averaged in 30-second bins. The isosbestic signal was fit to the GRAB_DA2m_ signal using a first-degree polynomial fit function. The fit isosbestic signal was then subtracted from the GRAB_DA2m_ signal to account for photobleaching and movement artifacts (dF/F). dF/F signal was z-scored to a five-minute baseline (from 6 min to 1 min before infusion) for comparison between rats (z dF/F). Area under the curve (AUC) for 30 min after infusion was calculated using the MATLAB function, trapz() and compared between groups. Peak z dF/F signal during the 30 min after the infusion was also compared between groups.

### 2.10. Histology

Rats were euthanized and transcardially perfused with 4 % paraformaldehyde (PFA). Brains were extracted and stored in PFA at 4 °C for 24 h and then in phosphate buffered saline and sucrose (20 %) at 4 °C for 48 h. To confirm fiber placement and viral expression, brains were frozen to − 20 °C for 20 min, sectioned into 20 μm coronal slices, and cover slipped with mounting media containing a DAPI stain.

### 2.11. Experiment 1: The effect of TMT on oxycodone IVSA

**Fig. 1.**
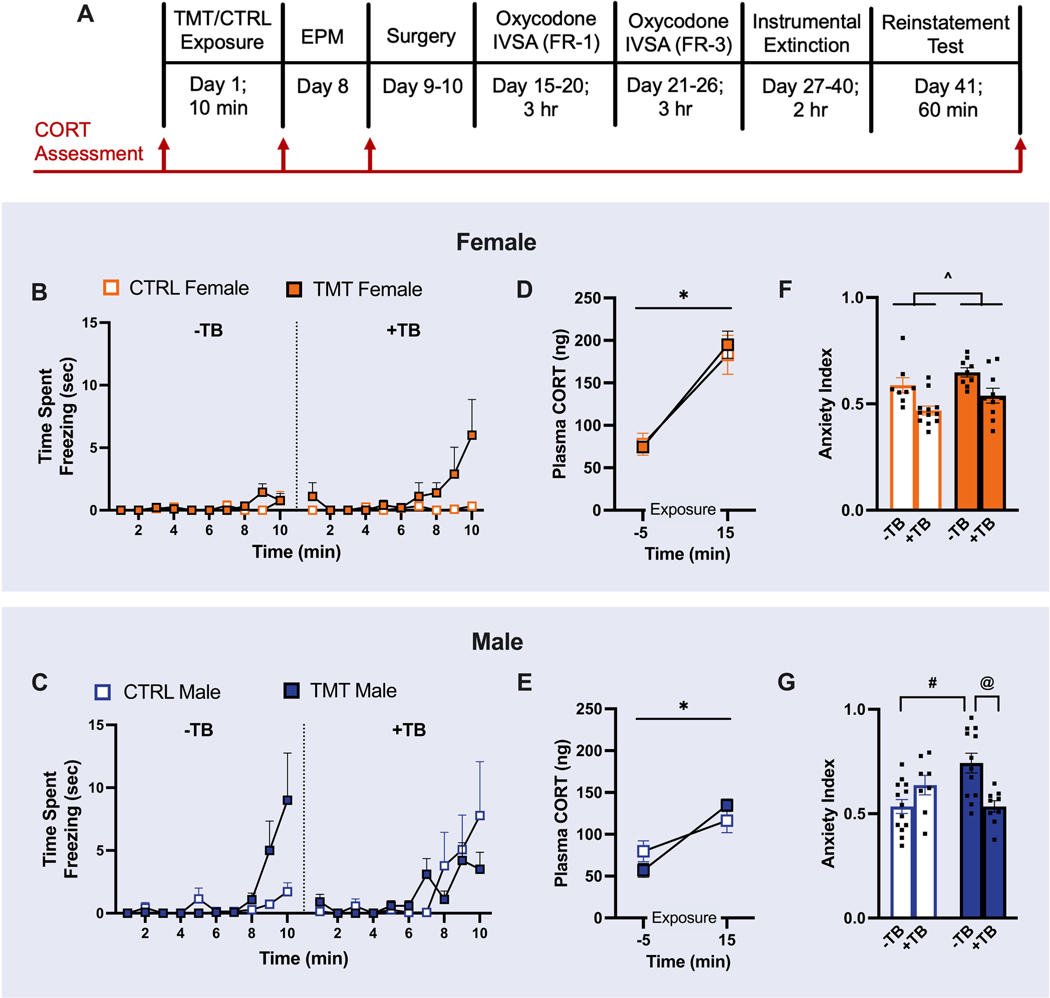
Both TMT exposure and the tail bleed procedure alter freezing and anxiety-like behavior in a sex-specific manner. A) Experiment 1 timeline. B) Amount of time spent freezing during exposure in female Control (CTRL; -TB *n* = 8, +TB *n* = 12) and TMT-exposed (-TB *n* = 9, +TB *n* = 10) rats. C) Amount of time spent freezing during exposure in male CTRL (-TB *n* = 7, +TB *n* = 14) and TMT-exposed (-TB *n* = 12, +TB *n* = 10) rats. D) In female rats that received tail bleeds, plasma CORT was increased following both CTRL (*n* = 9) and TMT (*n* = 10) exposure. E) Plasma CORT was also increased after CTRL (*n* = 7) and TMT (*n* = 9) exposure in male rats that received tail bleeds. F) TMT increased anxiety-like behavior on the EPM in female rats. A history of tail blood collection (+TB) reduced anxiety-like behavior in female rats in both TMT/Control conditions. G) TMT increased anxiety-like behavior in male rats which was prevented in the +TB condition. Mean ± SEM. * = main effect of Time *p* < 0.05; ^ = main effect of Group (TMT vs. CTRL) *p* < 0.05; # = CTRL -TB vs. TMT -TB *p* < 0.05; @ = TMT -TB vs. TMT +TB *p* < 0.05.

At approximately 9 weeks of age, 26 males and 27 females were exposed to TMT while 21 males and 22 females were exposed to the unscented control (CTRL) condition. One week later, rats were tested for anxiety-like behavior using EPM. Twenty-four hours later, CORT was assessed in a subset of rats (12 TMT males, 14 CTRL males, 13 TMT females, 14 CTRL females). In these rats, tail blood was collected at four time points: 5 min before and 15 min after TMT/control exposure, eight days after TMT/control exposure (24 h after EPM), and 2 h after the reinstatement test (Fig. 1A). The tail bleed procedure itself can be a source of stress that could interact with TMT to alter later anxiety-like behavior, and thus only a subset of rats received this procedure. One day after EPM, rats were surgically implanted with indwelling jugular catheters.

After recovery from catheter implantation surgery (5–6 days), rats underwent 12 oxycodone IVSA sessions, instrumental extinction, and reinstatement testing as described above. Immediately after the cue-primed reinstatement test, tail blood was collected for the measurement of CORT and rats were euthanized.

### 2.12. Experiment 2: The effect of TMT on nucleus accumbens core dopamine response to intravenous oxycodone in female rats

A separate cohort of female Sprague Dawley rats (*n* = 26) was used to investigate whether TMT exposure alters the dopamine response to IV oxycodone. To accomplish this, rats received jugular catheter surgery, viral vector infusion, and fiber implant surgery as described above. One week later, rats were exposed to TMT (*n* = 13) or the unscented control (*n* = 13) condition. One week later, rats were tested for anxiety-like behavior using EPM as described above.

One week after EPM and three weeks after surgery (akin to the time at which rats in Experiment 1 started IVSA; see Fig. 4a), rats underwent fiber photometry sessions as described above. Prior to these sessions, rats were habituated to the recording environment and vaginal lavage. On the first two days of habituation, rats were placed in a plexiglass activity chamber (16×16×15 in.) to be used for the recordings for 1 hour each day. On the third day of habituation, rats were placed into the chamber for 30 min while tethered to a fiber optic patch cord (400 μm diameter, 0.57 NA, Doric Lenses, Quebec, Canada) secured to implanted ferrules with a bronze mating sleeve (Thorlabs, Newton, NJ) and silicone tubing (Instech Laboratories, Plymouth Meeting, PA). On test days, rats were placed into the activity chamber for 10 min prior to recording. After 18 min of baseline recording, rats were infused with IV oxycodone (0, 0.25, or 0.5 mg/kg; 200 μl/min) such that all rats experienced each dose in a counterbalanced manner on different days with at least one day between recording sessions. Photometry signals were recorded for one hour after infusion. Locomotion was tracked throughout the session with Photobeam Activity Software (San Diego Instruments, San Diego, CA) and the total number of horizontal beam breaks was recorded to compare ambulation between groups.

### 2.13. Statistical analysis

Data were analyzed using GraphPad Prism software (v. 10.2.3; GraphPad, San Diego, CA) with an alpha level of 0.05. GLM assumptions were met prior to the use of parametric tests. Where data did not meet normality assumptions, data was log transformed prior to parametric analyses. Greenhouse-Geisser corrections were used to correct violations of sphericity [42]. The Grubbs test was used to identify outliers.

Percent change in CORT levels from 5 min before exposure to 15 min after was calculated and compared between groups. An anxiety index was calculated from dependent measures from the EPM, using the following equation: 1-[((time spent in the open arms/total time of test) + (number of open arm entries/total exploration on maze))/2] [43,44]. Time spent freezing, CORT (absolute and % change), anxiety index, time spent in the open arms (OA) and closed arms (CA) of the EPM, entries into the open and closed arms of EPM, EPM locomotion, active/inactive lever presses, infusions earned, and oxycodone intake were compared between groups and conditions using Student’s *t*-tests or two- or three-way ANOVAs with the factors Group (TMT/CTRL), Tail Bleed (±), and/or Time. Repeated measures (RM) were conducted on Time. Where necessary, these analyses were done separately by sex because prior research indicates sex differences in the behavioral impacts of TMT [45]. The AUC of the dopamine signal, locomotor activity after oxycodone infusion, and peak z dF/F signal 30 min after oxycodone infusion were compared between Dose (0, 0.25, 0.5 mg/kg) and Group (TMT/CTRL) using ANOVAs, with Dose or Time as within-subject variables as appropriate. In the case of missing values, mixed-effects ANOVAs were used; these instances are detailed below. Sidak’s post-hoc analyses were used to investigate significant interactions. To determine whether reinstatement of drug-seeking occurred in each group, the average number of lever presses during the last two days of extinction were compared to those during the test using a *t*-test. Spearman correlations were used to assess linear relationships between CORT, anxiety-like behavior, and self-administration, extinction, and reinstatement.

To probe whether anxiety-like behavior influences the relationship between drug-taking and -seeking in a continuous manner, multiple linear regressions (MLRs) were conducted with anxiety index and total oxycodone intake during self-administration as predictors of active lever pressing during the cue-induced reinstatement test. Since the tail bleed procedure influenced anxiety index, tail bleed condition was added as a categorical variable in the MLR. The same MLR model was used for TMT-exposed and unstressed control male and female rats separately: *Y = β*0 + *β*1(anxiety index) + *β*2(total oxycodone intake) + *β*3(tail bleed condition). Variance inflation factors (VIF) were used to check for multicollinearity with a standard that VIFs > 2 would indicate problematic multicollinearity that would impact model accuracy.

## 3. Results

### 3.1. Experiment 1

Rats that died prior to self-administration endpoints were excluded from all analyses (*n* = 3), with the exception of one male TMT-+ TB rat that was assessed for CORT. This rat died during surgery but was retained in analyses of dependent measures collected prior to death, due to the limited sample size assessed for CORT. Two rats did not acquire self-administration and were removed from analyses (1 female TMT -TB, 1 female TMT +TB). Five rats were excluded due to catheter failure (2 female TMT -TB, 1 female TMT +TB, 1 female CTRL +TB, and 1 male TMT +TB). Two rats failed to meet extinction criteria and were excluded from analyses (1 male TMT +TB and 1 female TMT +TB). One female TMT-exposed (-TB) rat was identified as an outlier across multiple dependent variables and was excluded from analyses. Therefore, analyses of self-administration variables were conducted on 42 male (CTRL -TB, *n* = 7; TMT -TB, *n* = 12; CTRL +TB, *n* = 14; TMT +TB, *n* = 9) and 39 female (CTRL -TB, *n* = 8; TMT -TB, *n* = 9; CTRL +TB, *n* = 12; TMT +TB, *n* = 10) rats. Three rats were removed from reinstatement analyses only due to illness (*n* = 1) and technical issues (*n* = 2).

#### 3.1.1. Freezing during TMT/control exposure

Female TMT-exposed rats increased time spent freezing during exposure relative to controls [Group x Time interaction: F _(9, 315)_ = 2.660, *p* = 0.0055; Fig. 1B]. There was a trend for a Group x Tail Bleed x Time interaction on time spent freezing in females [F _(9, 315)_ = 1.871, *p* = 0.0556]. A 3-way RM ANOVA indicated a Group x Tail Bleed x Time interaction for time spent freezing in male rats, with no significant multiple comparisons and seemingly opposing effects of TMT on freezing in the two Tail Bleed conditions [F _(9, 351)_ = 2.238, *p* = 0.0193; Fig. 1C]. See Supplemental Table 1 for additional statistical results.

#### 3.1.2. Corticosterone concentrations

To conserve resources, CORT analysis was conducted for a subset of TMT-exposed (*n* = 10) and control (*n* = 9) female rats and TMT-exposed (*n* = 9) and control (*n* = 7) male rats that did not differ from their larger cohort in anxiety index score. The baseline CORT concentrations of one male TMT-, one female TMT-, and one female CTRL-exposed rat were identified as outliers by Grubbs’ test and were excluded from analysis. Additionally, sufficient blood could not be collected from one male CTRL and one male TMT exposed rat after TMT-exposure. Therefore, mixed effects ANOVAs were used to assess group differences from baseline to after TMT/CTRL exposure in male (CTRL, *n* = 7; TMT, *n* = 9) and female (CTRL, *n* = 9; TMT, *n* = 10). CORT increased from baseline to 15 min after exposure regardless of Group in female [main effect of Time: F_(1, 31)_ = 49.72, *p* <0.001; Fig. 1D] and male rats [F _(1, 11)_ = 33.80, *p* = 0.0001; Fig. 1E]. No effect of Group [F _(1,31)_ = 0.07, *p* = 0.2917] or Time x Group [F _(1,31)_ = 0.2283, *p* = 0.6361] interaction on CORT was detected in female rats. Similarly, no main effect of Group [F _(1,14)_ = 0.06206, *p* = 0.8069] or Time x Group [F _(1,11)_ = 3.909, *p* = 0.0736] interaction was detected in male rats. Twenty-four hours after the EPM test, blood was collected for plasma CORT analysis in male (CTRL, *n* = 7; TMT, *n* = 9) and female (CTRL, *n* = 9; TMT, *n* = 8) rats. Sufficient blood could not be collected from two female TMT rats and were excluded from analysis. CORT did not differ between groups in female (CTRL, *mean* = 105.0, SEM = 7.6; TMT, *mean* = 113.9, SEM = 17.7; not shown) and male (CTRL, *mean* = 117.6, SEM = 23.6; TMT, *mean* = 126.3, SEM = 14.85; not shown) rats. Blood was also collected for CORT analysis 2 h after the start of the cue test and compared between males (CTRL, *n* = 6; TMT, *n* = 6) and females (CTRL, *n* = 8; TMT, *n* = 10). Sufficient blood could not be collected from two male TMT rats at this time and one control male and one control female rat were identified as outliers by Grubbs’ test and were excluded from analysis. There was no effect of Group on plasma CORT for either female (CTRL, *mean* = 41.9, SEM = 11.6; TMT, *mean* = 47.3, SEM = 7.6; not shown) or male (CTRL, *mean* = 31.3, SEM = 4.9; TMT, *mean* = 33.8, SEM = 6.3; not shown) rats. Additional statistical details can be found in Supplemental Table 2.

A positive correlation between baseline CORT and time spent in the open arms of EPM was identified in male [r _(6)_ = 0.7104, *p* = 0.0483; not shown], but not female rats exposed to TMT. No significant correlations were identified between CORT and any other behavioral measures assessed in TMT-exposed rats. In female and male control rats, there was no correlation between baseline CORT or post-exposure CORT and time spent freezing or anxiety-like behavior. There was also no relationship between the percent change in CORT from baseline to post-exposure and any fear or anxiety-related behavior, nor were there any significant correlations between CORT concentrations and lever pressing during the reinstatement test.

#### 3.1.3. Anxiety-like behavior

Anxiety index scores were greater in TMT-exposed female rats compared to CTRL-exposed females [main effect of Group: F _(1, 35)_ = 5.232, *p* = 0.0283; Fig. 1F]. A main effect of Tail Bleed on anxiety index was also identified [F _(1, 35)_ = 16.01, *p* = 0.0003], with tail bleeds conducted at the same time as TMT/CTRL exposure reducing anxiety index. A main effect of Group [F _(1,35)_ = 7.460, *p* = 0.0098] and a Group x Tail Bleed interaction [F _(1,35)_ = 4.572, *p* = 0.0396] were detected on locomotion during EPM (not shown). Sidak’s multiple comparisons showed that TMT reduced locomotion in female rats without tail bleeds (*p* = 0.0052), with no effect in female rats that received tail bleeds (*p* = 0.8820). Statistical results for all EPM measures in female rats are reported in Supplemental Table 3.

There was a significant Group x Tail Bleed interaction for the anxiety index in male rats [F _(1, 38)_ = 14.36, *p* = 0.0005; Fig. 1G]. Sidak’s post hoc analyses revealed that male TMT-exposed rats that did not receive a tail bleed prior to TMT exposure displayed an increased anxiety index compared to Controls without tail bleeds (*p* = 0.0003). Control and TMT-exposed male rats with tail bleeds did not differ in anxiety index (*p* = 0.1140). A non-significant trend for a main effect of Tail Bleed was detected in locomotion during EPM [F _(1,39)_ = 3.439, *p* = 0.0713], where the mean locomotion was greater in male rats that received tail bleeds compared to males that did not. No effect of Group [F _(1,39)_ = 0.65481, *p* = 0.4233] or Group x Tail Bleed interaction [F _(1,39)_ = 0.2944, *p* = 0.5905] was detected on EPM locomotion. Statistical results for all EPM measures in male rats are reported in Supplemental Table 4.

#### 3.1.4. Oxycodone IVSA

**Fig. 2.**
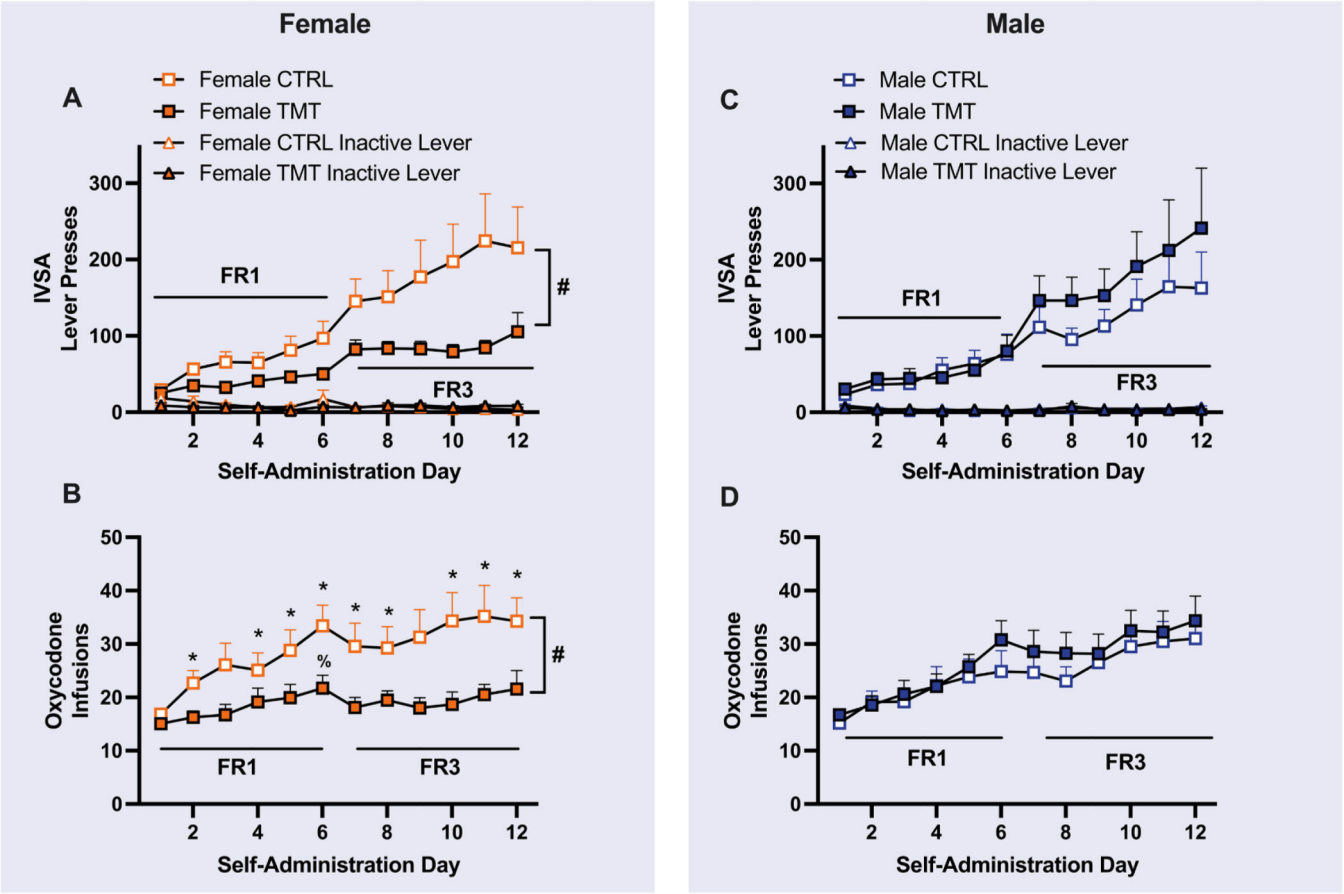
Predator scent stress produces long-lasting effects on oxycodone self-administration in female but not male rats. A) Female Control (CTRL; *n* = 20) and TMT (*n* = 19) rats increased active lever presses for oxycodone throughout self-administration. CTRL females displayed greater number of active lever presses than TMT rats, with no effect of TMT on inactive lever pressing. B) Female rats with a history of TMT exposure exhibited a reduction in the number of oxycodone infusions earned compared to CTRL. C) Male CTRL (*n* = 21) and TMT (*n* = 21) rats increased active lever pressing throughout self-administration regardless of stress condition. No group differences in inactive pressing were observed. D) Male rats increased the number of oxycodone infusions earned with no effect of TMT. Mean ± SEM. # = Group x Time interaction *p* < 0.05, * = *p* < 0.05 compared to CTRL Day 1, % = *p* < 0.05 compared to TMT Day 1.

Three-way ANOVAs did not identify a main effect or interaction with the Tail Bleed factor for any self-administration dependent variable in female or male rats (Supplemental Tables 5 and 6). Therefore, results are presented with both tail bleed and non-tail bleed conditions collapsed together in their respective Group (TMT or control). Throughout the 12 days of oxycodone IVSA, female rats increased the number of active lever responses for oxycodone [main effect of Time: F _(1.442, 53.36)_= 14.18, *p* < 0.0001; Fig. 2A], but not inactive lever responses [F _(1.607,_ 59.45)= 1.183, *p* = 0.3052; Fig. 2A]. A main effect of Group [F _(1,37)_ = 4.819, *p* = 0.0345] and a Group x Time interaction [F _(11,407)_ = 3.053, *p* = 0.0006] were found for active lever presses, such that TMT exposed rats displayed reduced active lever presses relative to CTRL throughout self-administration sessions. There was no effect of Group I [F _(1,37)_ = 0.3698, *p* = 0.5468] or Group x Time interaction [F _(11,407)_ = 1.046, *p* = 0.4045] for inactive lever presses. Significant effects of Time [F (2.902,107.4) = 6.634, *p* = 0.004], Group [F (1,37) = 6.712, *p* = 0.0136], and a Group x Time interaction [F _(11, 407)_ = 1.991, *p* = 0.0280] were detected for the number of infusions earned (Fig. 2B). Overall, TMT-exposed rats earned less infusions than CTRL-exposed rats but post-hoc tests did not find group differences for any IVSA day. Post-hoc tests comparing the number of infusions earned to Day 1 found that beginning on Day 2 and for several subsequent days, CTRL rats increased the number of infusions while TMT-exposed rats only increased infusions on Day 6. A significant Time x Group interaction was detected for oxycodone intake [F _(11, 407)_ = 2.008, *p* = 0.0264] with main effects of Time [F (3.072, 113.7) = 6.011, *p* = 0.0007] and Group [F (1,37) = 7.423, *p* = 0.0097], indicating that female rats reduced oxycodone intake over time compared to controls (not shown). Since rats received access to oxycodone on an FR1 for the first 6 days of IVSA followed by 6 days on an FR3, we also computed these analyses on each schedule of reinforcement separately and found the same results.

Throughout 12 days of IVSA, male rats increased active lever pressing [main effect of Time: F _(1.488, 55.06)_ = 14.42, *p* < 0.0001, Fig. 2C]. A main effect of Time was also detected for inactive lever presses [F _(4.069, 162.8)_ = 3.371, *p* = 0.0107; Fig. 2C). No effect of Group [F (1,40) = 0.5937, *p* = 0.4455] or Group x Time interaction [F (11,440) = 0.7833, *p* = 0.6570] was detected for the number of active lever presses. No effect of Group [F _(1,40)_ = 0.2502, *p* = 0.6197] or Group x Time interaction [F _(11,440)_ = 1.190, *p* = 0.2912] was detected for the number of inactive lever presses. There was a main effect of Time on the number of oxycodone infusions [F _(3.722, 189.9)_ = 14.65, *p* < 0.0001; Fig. 2D], but no effect of Group [F _(1,40)_ = 0.4242, *p* = 0.5186] or Group x Time interaction [F _(11,440)_ = 0.5077, *p* = 0.8983] on this measure. A significant effect of Time [F _(3.536, 141.5)_ = 12.55, *p* < 0.0001] on oxycodone intake (not shown) was detected, as male rats (TMT and Control) oxycodone intake throughout the 12 IVSA sessions. No effect of Group [F (1,40) = 0.3112, *p* = 0.5800] or Group x Time interaction [F (11,440) = 0.5997, *p* = 0.8294] was detected for oxycodone intake. When conducting these analyses on the FR-1 and FR-3 phases separately, the same results were found (not shown).

#### 3.1.5. Instrumental extinction and cue-induced reinstatement

**Fig. 3.**
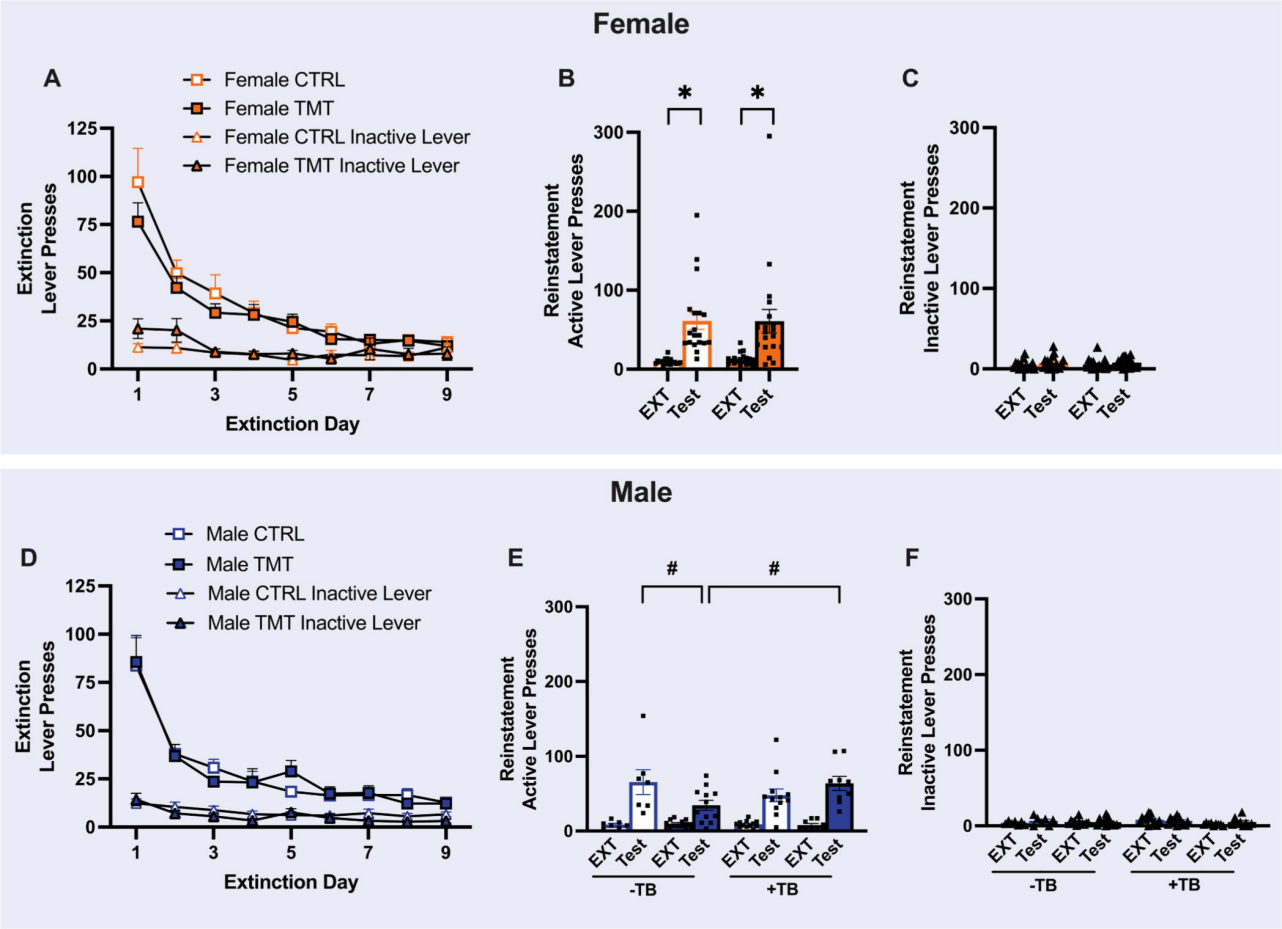
Predator scent stress did not alter instrumental extinction or cue-primed reinstatement of oxycodone-seeking. A) Female CTRL (*n* = 20) and TMT (*n* = 19) rats reduced active and inactive responses throughout instrumental extinction (main effect of Time) with no effect of TMT or a prior history of tail bleeds. B) Female CTRL (*n* = 20) and TMT (*n* = 19) rats reinstated oxycodone-seeking during a cue-induced reinstatement test regardless of stress history or a prior history of tail bleeds. C) Inactive lever presses were slightly increased during the reinstatement test in female rats. D) Male CTRL (*n* = 21) and TMT (*n* = 21) rats reduced active and inactive responses throughout instrumental extinction, with no effect of prior TMT exposure or prior history of tail bleeds on these measures. E) While a history of tail bleeds affected active lever pressing during extinction and reinstatement, male CTRL (*n* = 19) and TMT (*n* = 21) rats reinstated oxycodone-seeking regardless of stress and tail bleed history. Male TMT rats that did not receive tail bleeds displayed fewer active lever presses during the cue test than TMT rats that did receive tail bleeds and male CTRL rats that did not receive tail bleeds. F) A Time x Group x Tail Bleed interaction was identified in inactive responses during the reinstatement test in male rats. Mean ± SEM., * = Main effect of Time (EXT v. Test) *p* < 0.05, # = *p* < 0.05 compared to TMT^−^ TB.

Three-way ANOVAs did not identify a main effect or interaction with the Tail Bleed factor for any extinction or reinstatement dependent variable in female rats (Supplemental Table 7 & 8, respectively). Therefore, results are presented with both tail bleed and non-tail bleed conditions collapsed in their respective Group (TMT or control). Throughout instrumental extinction, female rats reduced active [F _(1.952,_ 72.23) = 37.30, *p* < 0.0001] and inactive lever presses [F (3.691, 136.6) = 4.850, *p* = 0.0015; Fig. 3A]. No effect of Group was detected for active [F (1, 37) = 0.6329, *p* = 0.4313] or inactive [F (1, 37) = 0.6321, *p* = 0.4317] lever presses. There were no significant Group x Time interactions in active [F (8296) = 0.9164, *p* = 0.5031] or inactive [F (8296) = 1.820, *p* = 0.0730] lever presses. A comparison of the number of active lever presses during the last two days of extinction (average) and during the cue test, revealed that both the TMT- and control-exposed groups reinstated active lever pressing during the test [main effect of Time: F_(1, 36)_ = 30.27, *p* = 0 < 0.0001, Fig. 3B]. No main effect of Group [F _(1, 36)_ = 0.0203, *p* = 0.8875] or Group x Time interaction [F _(1, 36)_ = 0.0346, *p* = 0.8535] was detected. Upon detection of a main effect of Time, and to confirm that reinstatement occurred, post-hoc tests comparing extinction to reinstatement were conducted. Female TMT (*p* = 0.0006) and female CTRL (*p* = 0.0012) rats reinstated oxycodone-seeking above extinction levels. Female rats also increased inactive lever presses during the test [F _(1, 36)_ = 4.627, *p* = 0.0383, Fig. 3C]. There was no effect of Group [F _(1, 36)_ = 0.2280, *p* = 0.6359] or a Group x Time interaction [F (1_, 36)_ = 0.0467, *p* = 0.8301] detected in inactive lever presses.

A three-way ANOVA did not identify a main effect or interaction with the Tail Bleed factor for active and inactive lever presses during extinction training in male rats (Supplemental Table 9) which are presented with both tail bleed and non-tail bleed conditions collapsed. Males decreased active lever pressing throughout extinction [main effect of Time: F _(1.922, 76.86)_ = 36.44, *p* < 0.0001, Fig. 3D] with no effect of Group [F _(1, 40)_ = 0.0003, *p* = 0.9865] or Group x Time interaction [F (8320) = 0.4283, *p* = 0.9038] detected. A main effect of Time was detected for inactive lever presses during extinction [F _(3.938, 157.5)_ = 11.58, *p* <0.0001, Fig. 3D], with no effect of Group [F _(1, 40)_ = 1.024, *p* = 0.3176] or Group x Time interaction detected [F _(8320)_ = 0.1.778, *p* = 0.0.0806]. A three-way ANOVA identified a significant Tail Bleed x Group x Test interaction in active [F_(1, 36)_ = 6.750, *p* = 0.0136] and inactive [F(1, 36) = 5.221, *p* = 0.0283] lever presses during extinction and the cue test in male rats (Supplemental Table 10). For active lever presses, Sidak’s post-hoc tests revealed that all groups (TMT and CTRL) and all tail bleed conditions (with and without) reinstated oxycodone-seeking above extinction levels. Male TMT rats that did not receive tail bleeds displayed fewer active lever presses during the cue test relative to TMT rats that did receive tail bleeds and to CTRL rats that did not receive tail bleeds. No significant post-hoc tests were found for inactive lever presses (Fig. 3F).

#### 3.1.6. Multiple linear regression analysis

In TMT-exposed female rats, the regression model significantly accounted for 54.04 % of the variance in active lever pressing during reinstatement [F _(3, 15)_ = 5.879, *p* = 0.0073] with anxiety index score (*β* = 282.5, SE = 128.7, *p* = 0.0444) and total oxycodone intake (*β* = 3.70, SE = 0.9691, *p* = 0.0015) emerging as significant predictors. In TMT-exposed male rats, the regression model significantly accounted for 40.49 % of the variance in active lever pressing during reinstatement [F (3_,17)_ = 3.855, *p* = 0.0284], however, none of the predictors reached significance. The same regression model was not significant in male and female unstressed controls.

### 3.2. Experiment 2

**Fig. 4.**
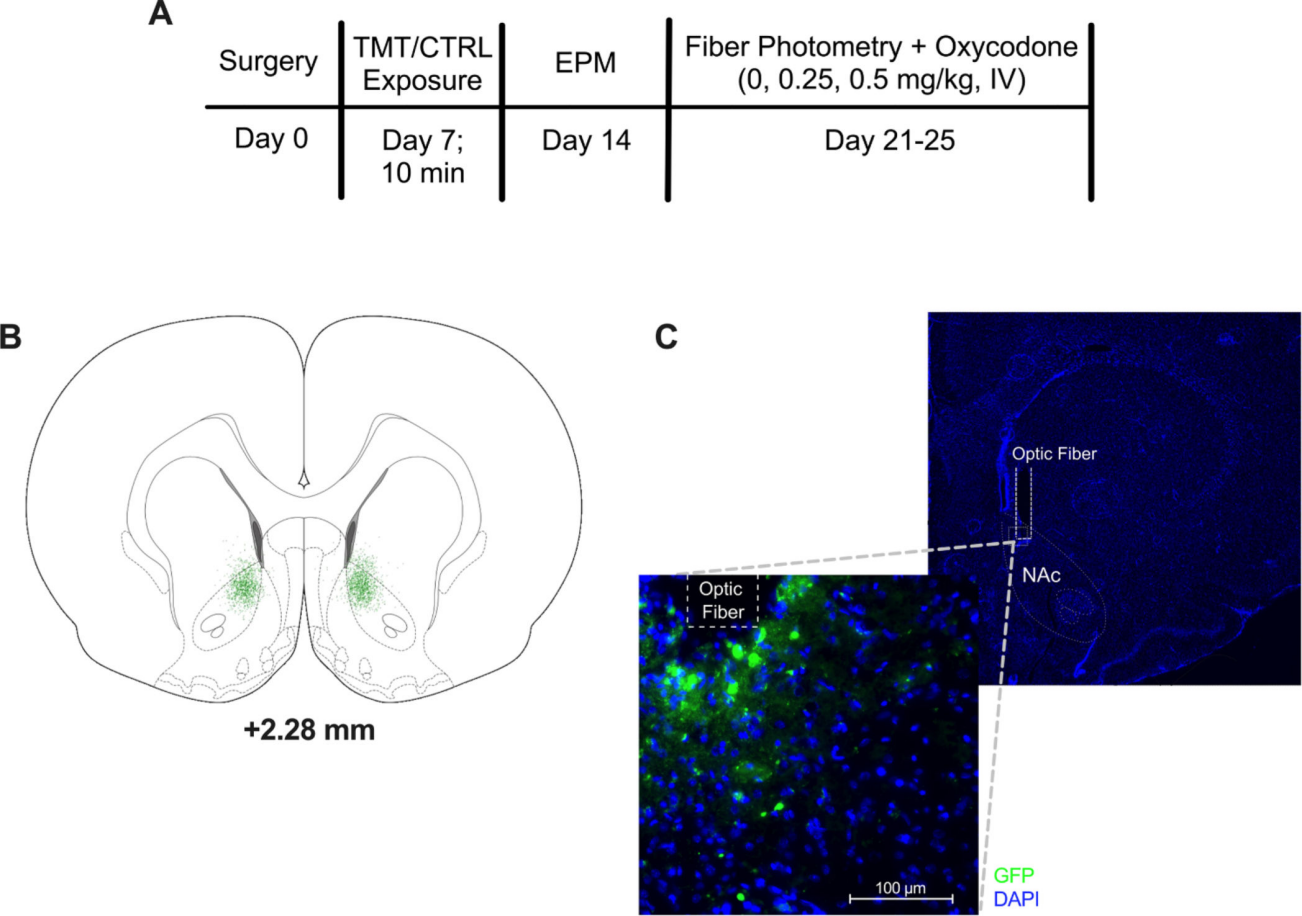
Experiment 2 timeline and representative images. A) Timeline. B) Schematic representation of viral spread in the nucleus accumbens core (NAc) with A/P relative to Bregma. C) Representative image of cannula placement and viral expression (GFP tag) beneath the optic fiber (40x).

To follow up on the strongest effects observed in Experiment 1, we investigated the long-term impact of TMT on oxycodone-induced dopamine dynamics in female rats only in Experiment 2. Histological assessment determined incorrect fiber placement for 3 control and 1 TMT rat, which were excluded from all analyses. One control rat lost its fiber implant before receiving its third oxycodone dose (0.5 mg/kg); therefore, mixed effects analyses were used as necessary. See Fig. 4B and C for a schematic representation of viral expression and representative images of optical fiber placement and viral expression.

#### 3.2.1 Anxiety-like behavior

**Fig. 5.**
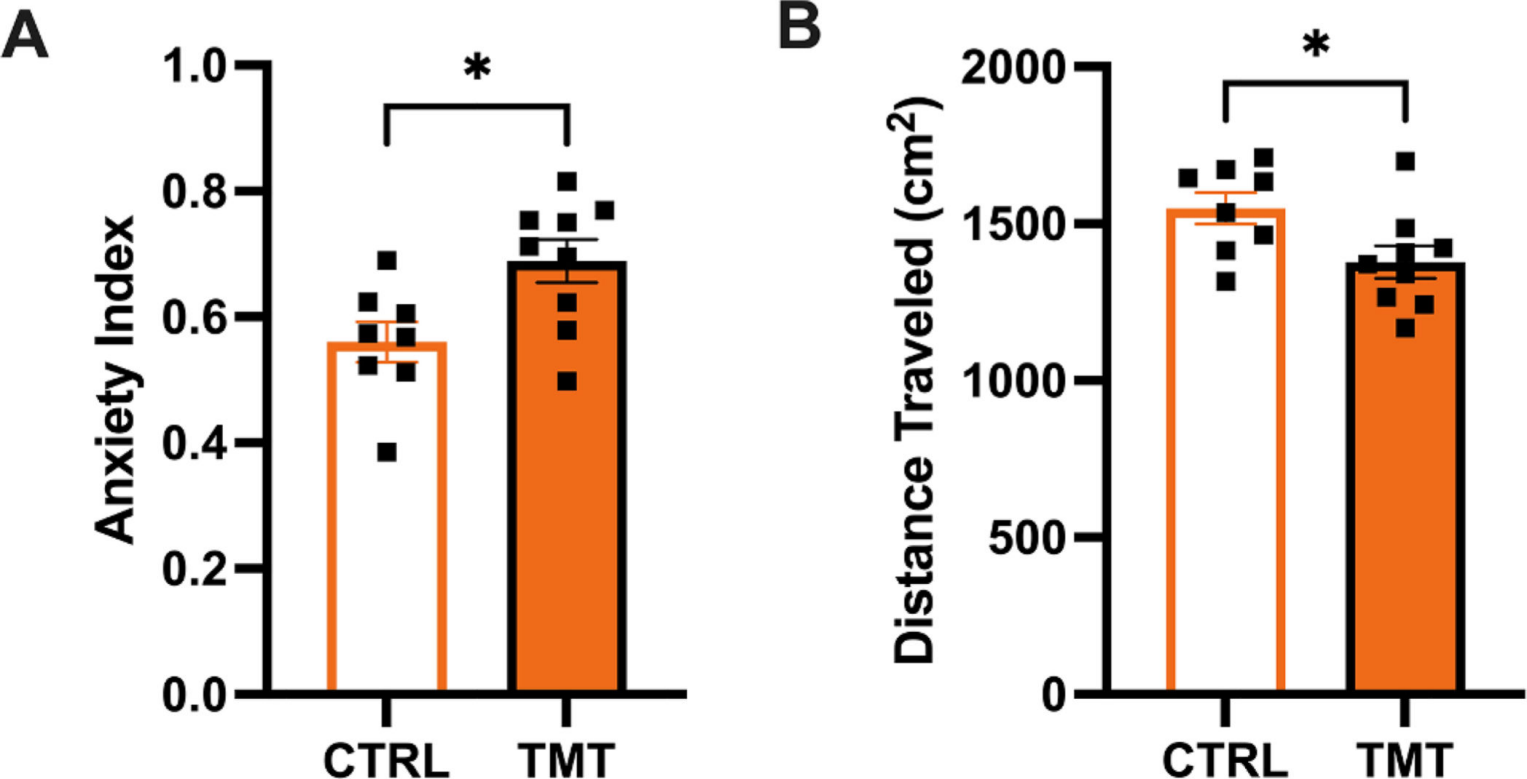
Effect of TMT on anxiety-like behavior and locomotion in Experiment 2. In the representative sample selected for fiber photometry, a history of TMT exposure A) increased anxiety index and B) reduced the distance traveled in the elevated plus maze (EPM) compared to unstressed controls (CTRL). Error bars = SEM. * = *p* < 0.05 CTRL vs. TMT.

There was no effect of TMT on anxiety index when considering all animals in Experiment 2 [t _(24)_ = 0.3043, *p* = 0.7636]. This may have occurred due to a lower sample size exposed to TMT in this experiment. To examine NAc DA dynamics in a representative sample of female rats with similar anxiety index scores to female rats in Experiment 1, 2 Control rats with high anxiety index scores and 3 TMT-exposed rats with low anxiety index score were excluded. Therefore, the remaining subset of rats (TMT *n* = 9; CTRL *n* = 8) demonstrated increased anxiety index scores after TMT exposure compared to control exposure [t _(16)_ = 2.262, *p* = 0.0380; Fig. 5A]. In this subset of rats, TMT exposure also reduced locomotor activity (distance traveled) compared to Control exposure [t (16) = 2.172, *p* = 0.0452; Fig. 5B]. The analyses of other EPM dependent measures are reported in Supplemental Table 11.

#### 3.2.2. Nucleus accumbens core dopamine response to oxycodone

**Fig. 6.**
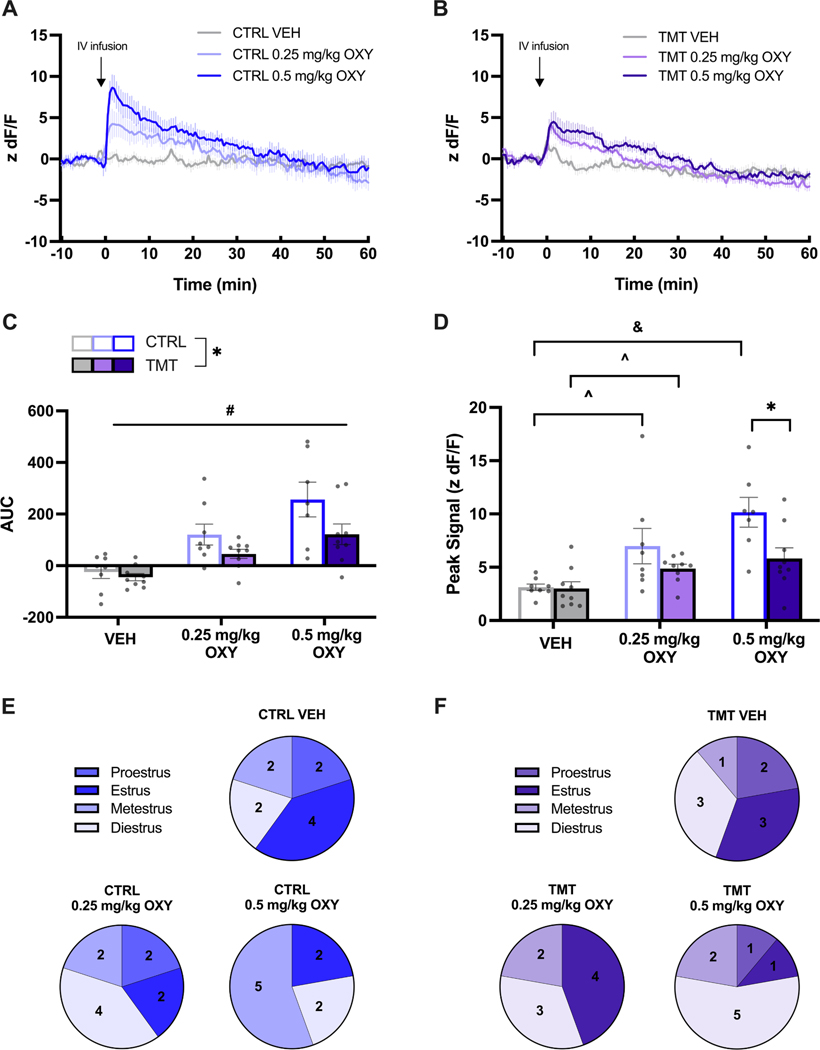
A history of TMT exposure reduces the nucleus accumbens core dopamine response to intravenous oxycodone. Average traces of nucleus accumbens core dopamine response to intravenous oxycodone (0, 0.25, and 0.5 mg/kg) in A) Control (*n* = 8) and B) TMT exposed (*n* = 9). female rats. C) A history of TMT exposure reduced the area under the curve signal for the 30 min after oxycodone (OXY) infusion compared to unstressed controls. D) Peak signal after OXY increased in a dose-dependent manner in Control (CTRL) rats. Peak signal increased between vehicle (VEH) to 0.25 mg/kg oxycodone and was reduced compared to Controls after 0.5 mg/kg OXY. Estrous phase during fiber photometry test sessions for E) CTRL and F) TMT-exposed rats. Error bars = SEM. * = *p* < 0.05 effect of Group, # = *p* < 0.05 effect of Dose, ^ = *p* < 0.05 VEH v. 0.25 mg/kg OXY, & = *p* < 0.05 VEH v. 0.5 mg/kg OXY.

Fiber photometry recordings were conducted for 10 min prior to and an hour after a single vehicle or oxycodone infusion (Fig. 6A and B), only in the subset of rats shown in Fig. 5A. A mixed effects analysis revealed main effects of Dose [F _(1.438, 20.85)_ = 19.71, *p* < 0.0001; Fig. 6C] and Group [F _(1, 15)_ = 6.478, *p* = 0.0224] on AUC DA, with the TMT-exposed group showing a reduced AUC DA relative to controls for the 30 min following oxycodone. No Dose x Group interaction was detected on DA AUC [F _(2, 29)_ = 1.315, *p* = 0.2841]. A significant effect of Dose [F _(1.812,_ 26.28) = 20.91, *p* < 0.0001], but not of Group [F (1, 15) = 3.973, *p* = 0.0648] and a significant Dose x Group interaction was detected for the peak DA signal after infusion [F _(2, 29)_ = 3.693, *p* = 0.0373; Fig. 6D]. Sidak’s post hoc tests revealed that TMT exposure reduced the z dF/F peak signal after a 0.5 mg/kg oxycodone infusion compared to Control rats (*p* = 0.0280). A significant increase from vehicle (VEH) to 0.25 mg/kg oxycodone (*p* = 0.0037) and a significant increase from VEH to 0.5 mg/kg oxycodone (*p* = 0.0367) was observed in Control rats. An increase from VEH to 0.25 mg/kg oxycodone was detected in TMT exposed rats (*p* = 0.0375). Rats were in various stages of the estrous cycle during recording sessions (Fig. 6E and F).

#### 3.2.3. Locomotor response to intravenous oxycodone in female rats

**Fig. 7.**
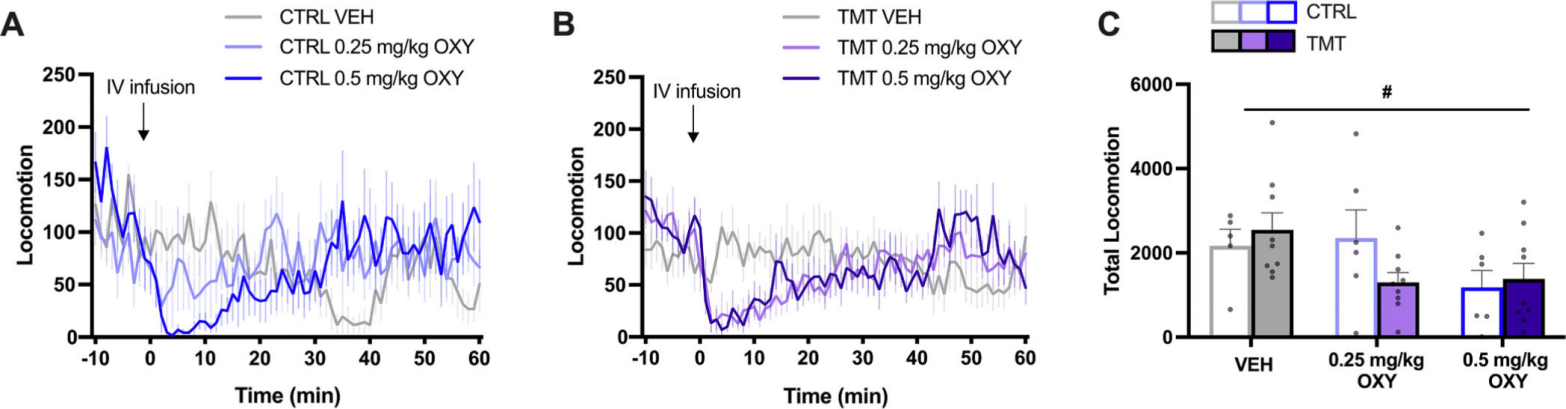
Locomotor response to intravenous oxycodone (OXY) or saline (VEH) for A) Control (CTRL) and B) TMT exposed female rats. C) Oxycodone reduced total locomotion for CTRL and TMT rats. Error bars = SEM. # = *p* < 0.05 effect of Dose.

Due to missing data from an equipment malfunction, a mixed effects analysis was used to compare total locomotor activity between groups across oxycodone doses. A main effect of Dose was identified [F _(1.375, 15.81)_ = 6.705, *p* = 0.0135] such that oxycodone decreased locomotor activity with no effect of Group or Dose x Group interaction (Fig. 7).

## 4. Discussion

In the present study, we investigated sex-specific effects of TMT exposure on plasma CORT concentrations, anxiety-like behavior, oxycodone self-administration and cue-induced reinstatement of oxycodone-seeking. We then assessed the impact of TMT exposure on the NA core dopamine response to intravenous oxycodone in female rats. TMT increased anxiety-like behavior in male and female rats that did not receive tail blood collection. In rats where tail blood was collected, CORT was elevated fifteen minutes after control and TMT exposure. Though the tail bleed procedure reduced anxiety-like behavior in TMT-exposed males and females, oxycodone self-administration was not affected by this procedure. Contrary to our initial hypotheses, we found that a history of TMT exposure reduced oxycodone intake exclusively in female rats, with no effects on active lever presses during extinction or a test for cued reinstatement. This effect was observed as early as Day 2 of self-administration. Fiber photometry recordings revealed that the reduction in intake may be due to an attenuation of oxycodone-evoked NA core DA following TMT exposure in female rats. Oxycodone self-administration and extinction learning did not differ between TMT or control-exposed male rats. Multiple linear regressions showed that, in TMT-exposed rats, the number of active lever presses during the cue-primed reinstatement test was strongly influenced by anxiety-like behavior and total oxycodone intake. This relationship was not observed in unstressed control rats, suggesting that TMT exposure has a lasting effect on reinstatement behavior weeks later. These findings suggest that TMT induces long-term adaptations in the NA core of female rats and/or changes to dopaminergic innervations to the NA core that contribute to reduced oxycodone intake during self-administration.

Lower baseline serum CORT was associated with higher anxiety-like behavior one week after TMT exposure in male rats, with no relationships identified in females. Others have reported that male rats with reduced amplitude of basal CORT pulsatility prior to PSS demonstrate the greatest anxiety-like behavior one week later [70], indicating that basal HPA-axis activity may increase stress-susceptibility. Consistent with these preclinical findings, decreased urinary, serum, and salivatory CORT after trauma is associated with a greater likelihood of PTSD diagnosis in humans [26–28]. Further, the risk for PTSD is greater when the qualifying event occurs at night, when CORT levels are low due to circadian rhythms [46]. Together, these findings suggest that lower CORT concentrations during trauma or stress exposure may contribute to more severe symptoms after stress exposure, however, our sample size was low and further investigations should assess a larger sample size.

We tested the hypothesis that a blunted CORT response to TMT would be associated with greater fear and anxiety-like behavior. However, CORT concentrations increased from baseline to post-exposure, regardless of TMT or control condition. Thus, the tail bleed procedure itself elevated circulating CORT in a manner that was not further increased by TMT, either due to a ceiling effect or to the collection at only one time point after exposure. Though this study did not directly compare male and female rats, the CORT response to TMT was higher in females than in males, as has been found for restraint stress [47,48]. Others have observed CORT concentrations to increase in response to tail bleeds and return to baseline 24 h later [18,49]. We did not observe any relationship between absolute or percent change in CORT levels after exposure and later fear or anxiety-like behavior. This relationship should be further investigated using a less invasive method of blood sampling.

Male and female TMT-exposed rats that did not undergo tail bleeds increased anxiety-like behavior relative to control rats one week later, indicating that TMT has long-lasting effects on anxiety-like behavior, as previously shown [18,25,31,32,44,50–52]. Anxiety-like behavior in EPM was attenuated in males that received tail bleeds. This is likely due to the increase in CORT following the tail bleed procedure, consistent with the literature that exogenous administration of CORT before or after PSS [29,30,52,53] and before acute immobilization stress [54] protects against the long-term effects of these stressors on anxiety-like behavior in male rodents. The protective effect of CORT treatment has also been observed in humans. The prevalence of PTSD is reduced at 6 months when hydrocortisone is administered prior to trauma exposure (cardiac surgery/septic shock) and when administered within six hours after trauma (accident or snake bite) [53].

We are the first to investigate the lasting effects of a single exposure to predator scent stress on oxycodone self-administration and reinstatement in male and female rats. In male rats, TMT exposure did not impact any oxycodone-seeking measure. In fact, TMT-exposed rats that received tail bleeds and displayed a reduced anxiety-index relative to TMT-exposed rats that did not receive a tail bleed showed increased cue-primed reinstatement. Thus, greater anxiety-like behavior in male rats following TMT does not appear to drive oxycodone-taking or seeking. Similarly, social defeat stress and restraint stress do not produce effects on heroin self-administration, breakpoint for heroin, or cue-primed reinstatement in male rats [55–57]. TMT exposure also has no effect on alcohol self-administration in male and female rats with passive stress responses to TMT [45]. Weeks after TMT exposure, cued cocaine- and methamphetamine‑seeking are increased [18,19]. Taken together, these findings indicate that the long-term effects of a single stress exposure on drug seeking in male rodents may be drug-specific, with increases in psychostimulant seeking and no effect on opioid and alcohol-seeking.

Contrary to our initial hypothesis, and unlike the effects observed in males, a history of TMT exposure reduced oxycodone intake in female rats relative to controls. Thus, female rats may have increased susceptibility to TMT-induced anhedonia and diminished reward sensitivity, in agreement with women with PTSD reporting greater symptoms of anhedonia than men [58,59]. To support this idea, following the same TMT-exposure procedure used here, we have found that female rats reduce sucrose intake weeks after a single exposure to TMT [25]. In contrast, male rodents exposed to predator stress do not reduce sucrose intake relative to controls [17,60]. After chronic social defeat stress, female rats that consume the least saccharin also self-administer the least cocaine [61], supporting a link between stress, anhedonia, and reward in female rodents. While it is also possible that reduced oxycodone intake in female rats after TMT exposure was due to greater sensitivity to oxycodone, this is unlikely in light of the reduced NA DA response to oxycodone observed here. Assessing changes in the dose response curve for oxycodone self-administration would provide additional clarity on this issue.

Weeks after TMT exposure, female, but not male, rats show increased DA turnover in the NA [24,62]. The rate of DA turnover negatively correlates with sucrose intake weeks after TMT exposure [24]. Therefore, upon finding decreased oxycodone self-administration following TMT in females, we hypothesized that TMT alters DA dynamics within the NA in female rats. Indeed, the NA DA response to oxycodone was blunted in TMT-exposed females, suggesting that reduced oxycodone self-administration after TMT was due to reduced, rather than enhanced, oxycodone reward. Notably, fiber photometry recordings can only quantify the change from baseline DA and not basal DA levels. It is possible that basal DA is also affected by TMT, such that if it is increased, less oxycodone-induced DA release is necessary for reward. However, the literature supports a hypodopaminergic state following TMT. Chronic inescapable stress (footshock stress and restraint) induces anhedonia accompanied by a reduced NA DA response to cocaine in male rats [63]. Repeated restraint stress results in increased DAT binding in the NA [64] and greater DAT density in the striatum is observed in humans with PTSD relative to unstressed and trauma-exposed controls [65]. Thus, it is possible that the present results are due to increased DAT activity. Since greater DA turnover is found in the NA after TMT exposure in female rats [24], it is possible that stress exposure increases monoamine oxidase (MAO) and catechol-O-methyltransferase resulting in greater DA degradation. Indeed, greater platelet MAO in individuals with PTSD has been associated with greater PTSD symptoms [66,67]. The data presented suggests that a reduced NAc DA response to oxycodone caused the reduction in oxycodone intake in female rats, however, we are unable to determine whether altered DA dynamics underlie the sex differences observed in Experiment 1, as males were not included in photometry experiments. Future work should further characterize sex differences in the effects of TMT on DA dynamics. Such work could potentially utilize a neutral scent as the control condition, to ensure that such effects do not generalize to odor-exposure alone.

Controls showed a dose-dependent increase in peak DA signal following oxycodone, however, TMT-exposed females increased peak signal from vehicle to 0.25 mg/kg, but not from 0.25 mg/kg oxycodone to 0.5 mg/kg oxycodone. While this may be due to TMT-induced changes in striatal DA signaling, it may also be due to the estrous phase at the time of testing. Estradiol increases basal NA DA [37], NA DA turnover [38], and enhances NA DA response to amphetamine [39]. Female mice in estrus demonstrate an increased basal VTA dopamine firing rate compared to females in diestrus [68] contributing to greater NA DA efflux in estrus. Here, a majority of TMT-exposed females received the lower dose (0.25 mg/kg) while in estrus, when NA DA is high, while for the highest dose (0.5 mg/kg), a majority of TMT-exposed females were in diestrus, a phase when NA DA is low (Fig. 6F). In fact, chronic restraint stress lengthens the duration of the estrous cycle in female mice and increases the number of days mice spend in diestrus [69].

## 5. Conclusions

Here we sought to characterize sex-specific effects of TMT on oxycodone self-administration and the underlying impacts on the mesolimbic dopamine system, finding that TMT exposure reduces both oxycodone intake and the DA response to intravenous oxycodone in female rats. Integrating these results with reports that TMT induces anhedonia and alters NA DA metabolism in female rats [24,25] suggests that TMT produces an enduring reduction in reward sensitivity in female rats that is driven by reduced NA core DA response to rewarding stimuli. A reduction in reward sensitivity is a potential mechanism by which stress exposure may render females more vulnerable to opioid abuse, requiring greater amounts of drug to achieve similar reinforcing effects. Additional work employing effort-based (e.g. progressive-ratio) and dose-response assessments of oxycodone-seeking are necessary to fully characterize the lasting effects of stress on opioid seeking.

## Supplementary Material

MMC1

Supplementary material associated with this article can be found, in the online version, at doi:10.1016/j.addicn.2025.100210.

## Data Availability

Data will be made available on request.

## References

[1] Kilpatrick DG, Resnick HS, Milanak ME, Miller MW, Keyes KM, Friedman MJ, National estimates of exposure to traumatic events and PTSD prevalence using DSM-IV and DSM-5 criteria, J. Trauma. Stress 26 (2013) 537–547, 10.1002/jts.21848. 24151000 PMC4096796

[2] Breslau N, Davis GC, Andreski P, Peterson E, Traumatic events and posttraumatic stress disorder in an urban population of young adults, Arch. Gen. Psychiatry 48 (1991) 216–222, 10.1001/archpsyc.1991.01810270028003. 1996917

[3] Na PJ, Schnurr PP, Pietrzak RH,Mental health of U.S. combat veterans by war era: results from the national health and Resilience in veterans study, J. Psychiatr. Res 158 (2023) 36–40, 10.1016/j.jpsychires.2022.12.019. 36565542 PMC11929138

[4] Olff M, Langeland W, Draijer N, Gersons BPR, Gender differences in posttraumatic stress disorder, Psychol. Bull 133 (2007) 183–204, 10.1037/0033-2909.133.2.183. 17338596

[5] Breslau N, Davis GC, Schultz LR, Posttraumatic stress disorder and the incidence of nicotine, alcohol, and other drug disorders in persons who have experienced trauma, Arch. Gen. Psychiatry 60 (2003) 289–294, 10.1001/archpsyc.60.3.289. 12622662

[6] Chilcoat HD, Breslau N, Investigations of causal pathways between ptsd and drug use disorders, Addict. Behav 23 (1998) 827–840, 10.1016/S0306-4603(98)00069-0. 9801719

[7] Kessler RC, Sonnega A, Bromet E, Hughes M, Nelson CB, Posttraumatic stress disorder in the National Comorbidity Survey, Arch. Gen. Psychiatry 52 (1995) 1048–1060, 10.1001/archpsyc.1995.03950240066012. 7492257

[8] Mills KL, Teesson M, Ross J, Peters L, Trauma, PTSD, and substance use disorders: findings from the Australian National Survey of Mental Health and Well-Being, Am. J. Psychiatry 163 (2006) 652–658, 10.1176/ajp.2006.163.4.652. 16585440

[9] Elman I, Borsook D, The failing cascade: comorbid post traumatic stress- and opioid use disorders, Neurosci. Biobehav. Rev 103 (2019) 374–383, 10.1016/j.neubiorev.2019.04.023. 31063739

[10] Brown PJ, Recupero PR, Stout R, PTSD substance abuse comorbidity and treatment utilization, Addict. Behav 20 (1995) 251–254, 10.1016/0306-4603(94)00060-3. 7484319

[11] Cohen H, Zohar J, Matar MA, Zeev K, Loewenthal U, Richter-Levin G, Setting apart the affected: the use of behavioral criteria in animal models of post traumatic stress disorder, Neuropsychopharmacology 29 (2004) 1962–1970, 10.1038/sj.npp.1300523. 15257304

[12] Cohen H, Kozlovsky N, Alona C, Matar MA, Joseph Z, Animal model for PTSD: from clinical concept to translational research, Neuropharmacology 62 (2012) 715–724, 10.1016/j.neuropharm.2011.04.023. 21565209

[13] Makhijani VH, Franklin JP, Van Voorhies K, Fortino B, Besheer J, The synthetically produced predator odor 2,5-dihydro-2,4,5-trimethylthiazoline increases alcohol self-administration and alters basolateral amygdala response to alcohol in rats, Psychopharmacology (Berl) 238 (2021) 67–82, 10.1007/s00213-020-05659-w. 32978649 PMC7796942

[14] Manjoch H, Vainer E, Matar M, Ifergane G, Zohar J, Kaplan Z, Cohen H, Predator-scent stress, ethanol consumption and the opioid system in an animal model of PTSD, Behav. Brain Res 306 (2016) 91–105, 10.1016/j.bbr.2016.03.009. 26965572

[15] Edwards S, Baynes BB, Carmichael CY, Zamora-Martinez ER, Barrus M, Koob GF, Gilpin NW, Traumatic stress reactivity promotes excessive alcohol drinking and alters the balance of prefrontal cortex-amygdala activity, Transl. Psychiatry 3 (2013) e296, 10.1038/tp.2013.70. 23982628 PMC3756295

[16] Brodnik ZD, Black EM, España RA, Accelerated development of cocaine-associated dopamine transients and cocaine use vulnerability following traumatic stress, Neuropsychopharmacology 45 (2020) 472–481, 10.1038/s41386-019-0526-1. 31539899 PMC6969179

[17] Brodnik ZD, Black EM, Clark MJ, Kornsey KN, Snyder NW, Españ RA, Susceptibility to traumatic stress sensitizes the dopaminergic response to cocaine and increases motivation for cocaine, Neuropharmacology 125 (2017) 295–307, 10.1016/j.neuropharm.2017.07.032. 28778834 PMC5585061

[18] Schwendt M, Shallcross J, Hadad NA, Namba MD, Hiller H, Wu L, Krause EG, Knackstedt LA, A novel rat model of comorbid PTSD and addiction reveals intersections between stress susceptibility and enhanced cocaine seeking with a role for mGlu5 receptors, Transl. Psychiatry 8 (2018) 209, 10.1038/s41398-018-0265-9. 30291225 PMC6173705

[19] Ferland CL, Reichel CM, McGinty JF, Effects of oxytocin on methamphetamine-seeking exacerbated by predator odor pre-exposure in rats, Psychopharmacology (Berl) 233 (2016) 1015–1024, 10.1007/s00213-015-4184-7. 26700240 PMC5003622

[20] Shorter D, Hsieh J, Kosten TR, Pharmacologic management of comorbid post-traumatic stress disorder and addictions, Am. J. Addict 24 (2015) 705–712, 10.1111/ajad.12306. 26587796

[21] Hassan AN, Le Foll B, Imtiaz S, Rehm J, The effect of post-traumatic stress disorder on the risk of developing prescription opioid use disorder: results from the National Epidemiologic Survey on Alcohol and Related Conditions III, Drug. Alcohol. Depend 179 (2017) 260–266, 10.1016/j.drugalcdep.2017.07.012. 28818717

[22] Cochran G, Bacci JL, Ylioja T, Hruschak V, Miller S, Seybert AL, Tarter R, Prescription opioid use: patient characteristics and misuse in community pharmacy, J. Am. Pharm. Assoc. (2003) 56 (2016) 248–256, 10.1016/j.japh.2016.02.012, e6. 27053277 PMC4886233

[23] Seal KH, Shi Y, Cohen G, Cohen BE, Maguen S, Krebs EE, Neylan TC, Association of mental health disorders with prescription opioids and high-risk opioid use in US veterans of Iraq and Afghanistan, JAMA 307 (2012) 940–947, 10.1001/jama.2012.234. 22396516

[24] Wilkinson CS, Blount HL, Schwendt M, Knackstedt LA, Brain monoamine dysfunction in response to predator scent stress accompanies stress-susceptibility in female rats, Biomolecules 13 (2023), 10.3390/biom13071055. PMC10377406 37509091

[25] Blount HL, Dee J, Wu L, Schwendt M, Knackstedt LA, Stress resilience-associated behaviors following predator scent stress are accompanied by upregulated nucleus accumbens mGlu5 transcription in female Sprague Dawley rats, Behav. Brain Res 436 (2023) 114090, 10.1016/j.bbr.2022.114090. 36057378

[26] McFarlane AC, Atchison M, Yehuda R, The acute stress response following motor vehicle accidents and its relation to PTSD, Ann. N. Y. Acad. Sci 821 (1997) 437–441, 10.1111/j.1749-6632.1997.tb48299.x. 9238224

[27] Delahanty DL, Raimonde AJ, Spoonster E, Initial posttraumatic urinary cortisol levels predict subsequent PTSD symptoms in motor vehicle accident victims, Biol. Psychiatry 48 (2000) 940–947, 10.1016/s0006-3223(00)00896-9. 11074232

[28] Ehring T, Ehlers A, Cleare AJ, Glucksman E, Do acute psychological and psychobiological responses to trauma predict subsequent symptom severities of PTSD and depression? Psychiatry Res 161 (2008) 67–75, 10.1016/j.psychres.2007.08.014. 18789538 PMC2943071

[29] Cohen H, Zohar J, Gidron Y, Matar MA, Belkind D, Loewenthal U, Kozlovsky N, Kaplan Z, Blunted HPA axis response to stress influences susceptibility to posttraumatic stress response in rats, Biol. Psychiatry 59 (2006) 1208–1218, 10.1016/j.biopsych.2005.12.003. 16458266

[30] Cohen H, Matar MA, Buskila D, Kaplan Z, Zohar J, Early post-stressor intervention with high-dose corticosterone attenuates posttraumatic stress response in an animal model of posttraumatic stress disorder, Biol. Psychiatry 64 (2008) 708–717, 10.1016/j.biopsych.2008.05.025. 18635156

[31] Shallcross J, Hámor P, Bechard AR, Romano M, Knackstedt L, Schwendt M, The divergent effects of CDPPB and cannabidiol on fear extinction and anxiety in a predator scent stress model of PTSD in rats, Front. Behav. Neurosci 13 (2019) 91, 10.3389/fnbeh.2019.00091. 31133832 PMC6523014

[32] Shallcross J, Wu L, Wilkinson CS, Knackstedt LA, Schwendt M, Increased mGlu5 mRNA expression in BLA glutamate neurons facilitates resilience to the long-term effects of a single predator scent stress exposure, Brain Struct. Funct 226 (2021) 2279–2293, 10.1007/s00429-021-02326-4. 34175993 PMC10416208

[33] Castañeda TR, de Prado BM, Prieto D, Mora F, Circadian rhythms of dopamine, glutamate and GABA in the striatum and nucleus accumbens of the awake rat: modulation by light, J. Pineal Res 36 (2004) 177–185, 10.1046/j.1600-079x.2003.00114.x. 15009508

[34] Pellow S, Chopin P, File SE, Briley M, Validation of open:closed arm entries in an elevated plus-maze as a measure of anxiety in the rat, J. Neurosci. Methods 14 (1985) 149–167, 10.1016/0165-0270(85)90031-7. 2864480

[35] Sun F, Zhou J, Dai B, Qian T, Zeng J, Li X, Zhuo Y, Zhang Y, Wang Y, Qian C, Tan K, Feng J, Dong H, Lin D, Cui G, Li Y, Next-generation GRAB sensors for monitoring dopaminergic activity in vivo, Nat. Methods 17 (2020) 1156–1166, 10.1038/s41592-020-00981-9. 33087905 PMC7648260

[36] Altshuler RD, Yang ES, Garcia KT, Davis IR, Olaniran A, Haile M, Razavi S, Li X, Role of orbitofrontal cortex in incubation of oxycodone craving in male rats, Addict. Biol 26 (2021) e12927, 10.1111/adb.12927. 32570285

[37] Espinosa E, Curtis KS, Increased locomotor activity in estrogen-treated ovariectomized rats is associated with nucleus accumbens dopamine and is not reduced by dietary sodium deprivation, Integr. Zool 13 (2018) 783–794, 10.1111/1749-4877.12333. 29851282

[38] Di Paolo T, Rouillard C, Bédard P, 17β-estradiol at a physiological dose acutely increases dopamine turnover in rat brain, Eur. J. Pharmacol 117 (1985) 197–203, 10.1016/0014-2999(85)90604-1. 4076343

[39] Becker JB, Estrogen rapidly potentiates amphetamine-induced striatal dopamine release and rotational behavior during microdialysis, Neurosci. Lett 118 (1990) 169–171, 10.1016/0304-3940(90)90618-j. 2125712

[40] McLean AC, Valenzuela N, Fai S, Bennett SAL, Performing vaginal lavage, crystal violet staining, and vaginal cytological evaluation for mouse estrous cycle staging identification, J. Vis. Exp (2012) e4389, 10.3791/4389. 23007862 PMC3490233

[41] Barker DJ, Miranda-Barrientos J, Zhang S, Root DH, Wang H-L, Liu B, Calipari ES, Morales M, Lateral preoptic control of the Lateral habenula through convergent glutamate and GABA transmission, Cell Rep 21 (2017) 1757–1769, 10.1016/j.celrep.2017.10.066. 29141211 PMC5699228

[42] Greenhouse SW, Geisser S, On methods in the analysis of profile data, Psychometrika 24 (1959) 95–112, 10.1007/BF02289823.

[43] Mazor A, Matar MA, Kaplan Z, Kozlovsky N, Zohar J, Cohen H, Gender-related qualitative differences in baseline and post-stress anxiety responses are not reflected in the incidence of criterion-based PTSD-like behaviour patterns, World J. Biol. Psychiatry 10 (2009) 856–869, 10.1080/15622970701561383. 17886167

[44] Cohen H, Geva AB, Matar MA, Zohar J, Kaplan Z, Post-traumatic stress behavioural responses in inbred mouse strains: can genetic predisposition explain phenotypic vulnerability? Int. J. Neuropsychopharmacol 11 (2008) 331–349, 10.1017/S1461145707007912. 17655807

[45] Ornelas LC, Tyler RE, Irukulapati P, Paladugu S, Besheer J, Increased alcohol self-administration following exposure to the predator odor TMT in active coping female rats, Behav. Brain Res 402 (2021) 113068, 10.1016/j.bbr.2020.113068. 33333108 PMC7882035

[46] Carmi L, Zohar J, Weissman T, Juven-Wetzler A, Bierer L, Yehuda R, Cohen H, Hydrocortisone in the emergency department: a prospective, double-blind, randomized, controlled posttraumatic stress disorder study. Hydrocortisone during golden hours, CNS Spectr (2022) 1–7, 10.1017/S1092852922000852. 35678177

[47] Haleem DJ, Kennett G, Curzon G, Adaptation of female rats to stress: shift to male pattern by inhibition of corticosterone synthesis, Brain Res 458 (1988) 339–347, 10.1016/0006-8993(88)90476-3. 2463050

[48] Aloisi AM, Ceccarelli I, Lupo C, Behavioural and hormonal effects of restraint stress and formalin test in male and female rats, Brain Res. Bull 47 (1998) 57–62, 10.1016/S0361-9230(98)00063-X. 9766390

[49] Tuli JS, Smith JA, Morton DB, Corticosterone, adrenal and spleen weight in mice after tail bleeding, and its effect on nearby animals, Lab. Anim 29 (1995) 90–95, 10.1258/002367795780740339. 7707684

[50] Nahum K, Todder D, Zohar J, Cohen H, The role of microglia in the (mal) adaptive response to traumatic experience in an animal model of PTSD, Int. J. Mol. Sci 23 (2022), 10.3390/ijms23137185. PMC9266429 35806185

[51] Hoffman JR, Ostfeld I, Kaplan Z, Zohar J, Cohen H, Exercise enhances the behavioral responses to acute stress in an animal model of PTSD, Med. Sci. Sports Exerc 47 (2015) 2043–2052, 10.1249/MSS.0000000000000642. 25699481

[52] Daskalakis NP, Cohen H, Cai G, Buxbaum JD, Yehuda R, Expression profiling associates blood and brain glucocorticoid receptor signaling with trauma-related individual differences in both sexes, Proc Natl Acad Sci USA 111 (2014) 13529–13534, 10.1073/pnas.1401660111. 25114262 PMC4169965

[53] Zohar J, Yahalom H, Kozlovsky N, Cwikel-Hamzany S, Matar MA, Kaplan Z, Yehuda R, Cohen H, High dose hydrocortisone immediately after trauma may alter the trajectory of PTSD: interplay between clinical and animal studies, Eur. Neuropsychopharmacol 21 (2011) 796–809, 10.1016/j.euroneuro.2011.06.001. 21741804

[54] Rao RP, Anilkumar S, McEwen BS, Chattarji S, Glucocorticoids protect against the delayed behavioral and cellular effects of acute stress on the amygdala, Biol. Psychiatry 72 (2012) 466–475, 10.1016/j.biopsych.2012.04.008. 22572034 PMC3753225

[55] Carter JS, Kearns AM, Vollmer KM, Garcia-Keller C, Weber RA, Baker NL, Kalivas PW, Reichel CM, Long-term impact of acute restraint stress on heroin self-administration, reinstatement, and stress reactivity, Psychopharmacology (Berl) 237 (2020) 1709–1721, 10.1007/s00213-020-05486-z. 32125483 PMC7857092

[56] Carter JS, Kearns AM, Reichel CM, Complex interactions between sex and stress on heroin seeking, Front. Neurosci 15 (2021) 784365, 10.3389/fnins.2021.784365. 34955731 PMC8702641

[57] Cruz FC, Quadros IM, Hogenelst K, Planeta CS, Miczek KA, Social defeat stress in rats: escalation of cocaine and “speedball” binge self-administration, but not heroin, Psychopharmacology (Berl) 215 (2011) 165–175, 10.1007/s00213-010-2139-6. 21197616 PMC3707112

[58] Nawijn L, van Zuiden M, Frijling JL, Koch SBJ, Veltman DJ, Olff M, Reward functioning in PTSD: a systematic review exploring the mechanisms underlying anhedonia, Neurosci. Biobehav. Rev 51 (2015) 189–204, 10.1016/j.neubiorev.2015.01.019. 25639225

[59] Mehta ND, Stevens JS, Li Z, Gillespie CF, Fani N, Michopoulos V, Felger JC, Inflammation, reward circuitry and symptoms of anhedonia and PTSD in trauma-exposed women, Soc. Cogn. Affect. Neurosci 15 (2020) 1046–1055, 10.1093/scan/nsz100. 32291455 PMC7657453

[60] Calvo-Torrent A, Brain PF, Martinez M, Effect of predatory stress on sucrose intake and behavior on the plus-maze in male mice, Physiol. Behav 67 (1999) 189–196, 10.1016/s0031-9384(99)00051-7. 10477049

[61] Shimamoto A, Holly EN, Boyson CO, DeBold JF, Miczek KA, Individual differences in anhedonic and accumbal dopamine responses to chronic social stress and their link to cocaine self-administration in female rats, Psychopharmacology (Berl) 232 (2015) 825–834, 10.1007/s00213-014-3725-9. 25178816 PMC4310791

[62] Morrow BA, Redmond AJ, Roth RH, Elsworth JD, The predator odor TMT, displays a unique, stress-like pattern of dopaminergic and endocrinological activation in the rat, Brain Res 864 (2000) 146–151, 10.1016/s0006-8993(00)02174-0. 10793199

[63] Scheggi S, Leggio B, Masi F, Grappi S, Gambarana C, Nanni G, Rauggi R, De Montis MG, Selective modifications in the nucleus accumbens of dopamine synaptic transmission in rats exposed to chronic stress, J. Neurochem 83 (2002) 895–903, 10.1046/j.1471-4159.2002.01193.x. 12421362

[64] Copeland BJ, Neff NH, Hadjiconstantinou M, Enhanced dopamine uptake in the striatum following repeated restraint stress, Synapse 57 (2005) 167–174, 10.1002/syn.20169. 15945060

[65] Hoexter MQ, Fadel G, Felício AC, Calzavara MB, Batista IR, Reis MA, Shih MC, Pitman RK, Andreoli SB, Mello MF, Mari JJ, Bressan RA, Higher striatal dopamine transporter density in PTSD: an in vivo SPECT study with [(99m) Tc]TRODAT-1, Psychopharmacology (Berl) 224 (2012) 337–345, 10.1007/s00213-012-2755-4. 22700036

[66] Pivac N, Knezevic J, Kozaric-Kovacic D, Dezeljin M, Mustapic M, Rak D, Matijevic T, Pavelic J, Muck-Seler D, Monoamine oxidase (MAO) intron 13 polymorphism and platelet MAO-B activity in combat-related posttraumatic stress disorder, J. Affect. Disord 103 (2007) 131–138, 10.1016/j.jad.2007.01.017. 17289152

[67] Svob Strac D, Petrovic ZK, Nikolac Perkovic M, Umolac D, Nedic Erjavec G, Pivac N, Platelet monoamine oxidase type B, MAOB intron 13 and MAOA-uVNTR polymorphism and symptoms of post-traumatic stress disorder, Stress 19 (2016) 362–373, 10.1080/10253890.2016.1174849. 27112218

[68] Calipari ES, Juarez B, Morel C, Walker DM, Cahill ME, Ribeiro E, Roman-Ortiz C, Ramakrishnan C, Deisseroth K, Han M-H, Nestler EJ, Dopaminergic dynamics underlying sex-specific cocaine reward, Nat. Commun 8 (2017) 13877, 10.1038/ncomms13877. 28072417 PMC5234081

[69] Breen KM, Thackray VG, Hsu T, Mak-McCully RA, Coss D, Mellon PL, Stress levels of glucocorticoids inhibit lhβ-subunit gene expression in gonadotrope cells, Mol. Endocrinol 26 (2012) 1716–1731, 10.1210/me.2011-1327. 22851703 PMC3458227

[70] Danan D, Matar MA, J Zohar Z, Cohen H, Blunted basal corticosterone pulsatility predicts post-exposure susceptibility to PTSD phenotype in rats, Psychoneuroendocrinology 87 (2018) 35–42, 10.1016/j.psyneuen.2017.09.023. 29035710

