# Supplementary material for: Predator scent stress reduces oxycodone self-administration and the nucleus accumbens dopamine response to oxycodone in female rats
Source: Addict Neurosci. Author manuscript; Available in PMC 2026 Feb 6. (PMC12875285; doi:10.1016/j.addicn.2025.100210)
Supplement: MMC1 [file NIHMS2115993-supplement-MMC1.docx]

**Table 1.** Results of 3-way ANOVAs on time spent freezing during TMT/CTRL exposure.

| **Female** | F (DFn, DFd) | P value |
| --- | --- | --- |
| Time | F (1.655, 57.92) = 3.889 | P=0.0332 |
| Group | F (1, 35) = 3.666 | P=0.0637 |
| Tail Bleed | F (1, 35) = 2.016 | P=0.1645 |
| Time x Group | F (9, 315) = 2.660 | P=0.0055 |
| Time x Tail Bleed | F (9, 315) = 1.389 | P=0.1919 |
| Group x Tail Bleed | F (1, 35) = 2.297 | P=0.1386 |
| Time x Group x Tail Bleed | F (9, 315) = 1.871 | P=0.0556 |
|  |  |  |
| **Male** | F (DFn, DFd) | P value |
| Time | F (1.657, 64.61) = 6.785 | P=0.0037 |
| Group | F (1, 39) = 0.2226 | P=0.6397 |
| Tail Bleed | F (1, 39) = 0.6455 | P=0.4266 |
| Time x Group | F (9, 351) = 0.4095 | P=0.9299 |
| Time x Tail Bleed | F (9, 351) = 0.2973 | P=0.9753 |
| Group x Tail Bleed | F (1, 39) = 0.9678 | P=0.3313 |
| Time x Group x Tail Bleed | F (9, 351) = 2.238 | P=0.0193 |

**Table 2.** Results of Student’s t-tests comparing CORT concentrations between TMT and CTRL conditions.

| **8 days after Exposure** | t (df) | P value |
| --- | --- | --- |
| Female _(CTRL: n = 9, TMT: n = 8)_ | t (15) = 0.4803 | P=0.6380 |
| Male _(CTRL: n = 7, TMT: n = 9)_ | t (14) = 0.3249 | P=0.7500 |
|  |  |  |
| **2-hrs after Reinstatement Test** |  |  |
| Female _(CTRL: n = 8, TMT: n = 10)_ | t (16) = 0.729 | P=0.1031 |
| Male _(CTRL: n = 6, TMT: n = 6)_ | t (10) = 0.1749 | P=0.8646 |

**Table 3.** Results of 2-way ANOVAs for the elevated plus maze measures in female rats.

| **Anxiety Index** | F (DFn, DFd) | P value |
| --- | --- | --- |
| Interaction | F (1, 35) = 0.02928 | P=0.8651 |
| Tail Bleed | F (1, 35) = 16.01 | P=0.0003 |
| Group | F (1, 35) = 5.232 | P=0.0283 |
|  |  |  |
| **Time in Open Arms** | F (DFn, DFd) | P value |
| Interaction | F (1, 35) = 0.3720 | P=0.5459 |
| Tail Bleed | F (1, 35) = 16.98 | P=0.0002 |
| Group | F (1, 35) = 14.23 | P=0.0006 |
|  |  |  |
| **Time in Closed Arms** | F (DFn, DFd) | P value |
| Interaction | F (1, 35) = 0.07734 | P=0.7826 |
| Tail Bleed | F (1, 35) = 3.445 | P=0.0719 |
| Group | F (1, 35) = 9.231 | P=0.0045 |
|  |  |  |
| **Open Entries** | F (DFn, DFd) | P value |
| Interaction | F (1, 35) = 0.001362 | P=0.9708 |
| Tail Bleed | F (1, 35) = 0.07805 | P=0.7816 |
| Group | F (1, 35) = 1.627 | P=0.2105 |
|  |  |  |
| **Closed arm entries** | F (DFn, DFd) | P value |
| Interaction | F (1, 35) = 1.576 | P=0.2176 |
| Tail Bleed | F (1, 35) = 16.44 | P=0.0003 |
| Group | F (1, 35) = 1.484 | P=0.2313 |
|  |  |  |
| **Locomotion** | F (DFn, DFd) | P value |
| Interaction | F (1, 35) = 4.572 | P=0.0396 |
| Tail Bleed | F (1, 35) = 0.07320 | P=0.7883 |
| Group | F (1, 35) = 7.460 | P=0.0098 |

**Table 4.** Results of two-way ANOVAs for the elevated plus maze parameters in male rats.

| **Anxiety Score** | F (DFn, DFd) | P value |
| --- | --- | --- |
| Interaction | F (1, 39) = 16.08 | P=0.0003 |
| Tail Bleed | F (1, 39) = 2.169 | P=0.1488 |
| Group | F (1, 39) = 1.326 | P=0.2565 |
|  |  |  |
| **Time in Open Arms** | F (DFn, DFd) | P value |
| Interaction | F (1, 39) = 1.769 | P=0.1912 |
| Tail Bleed | F (1, 39) = 14.43 | P=0.0005 |
| Group | F (1, 39) = 0.5457 | P=0.4645 |
|  |  |  |
| **Time in Closed Arms** | F (DFn, DFd) | P value |
| Interaction | F (1, 39) = 2.193 | P=0.1466 |
| Tail Bleed | F (1, 39) = 2.570 | P=0.1170 |
| Group | F (1, 39) = 2.486 | P=0.1230 |
|  |  |  |
| **Open Entries** | F (DFn, DFd) | P value |
| Interaction | F (1, 39) = 0.4918 | P=0.4873 |
| Tail Bleed | F (1, 39) = 0.5647 | P=0.4569 |
| Group | F (1, 39) = 1.377 | P=0.2478 |
|  |  |  |
| **Closed arm entries** | F (DFn, DFd) | P value |
| Interaction | F (1, 39) = 0.3706 | P=0.5462 |
| Tail Bleed | F (1, 39) = 19.16 | P<0.0001 |
| Group | F (1, 39) = 0.9980 | P=0.3240 |
|  |  |  |
| **Locomotion** | F (DFn, DFd) | P value |
| Interaction | F (1, 39) = 0.2944 | P=0.5905 |
| Tail Bleed | F (1, 39) = 3.439 | P=0.0713 |
| Group | F (1, 39) = 0.6548 | P=0.4233 |

**Table 5.** Results of 3-way ANOVAs on active lever pressing, inactive lever pressing, infusions, and intake during self-administration in female rats.

| **Active Lever** | F (DFn, DFd) | P value |
| --- | --- | --- |
| Time | F (1.422, 49.76) = 13.80 | P=0.0001 |
| Group | F (1, 35) = 4.355 | P=0.0443 |
| Tail Bleed | F (1, 35) = 0.005447 | P=0.9416 |
| Time x Group | F (11, 385) = 3.092 | P=0.0005 |
| Time x Tail Bleed | F (11, 385) = 0.2188 | P=0.9963 |
| Group x Tail Bleed | F (1, 35) = 0.06843 | P=0.7952 |
| Time x Group x Tail Bleed | F (11, 385) = 0.3159 | P=0.9823 |
|  |  |  |
| **Inactive Lever** | F (DFn, DFd) | P value |
| Time | F (1.609, 56.33) = 0.8260 | P=0.4198 |
| Group | F (1, 35) = 0.1205 | P=0.7306 |
| Tail Bleed | F (1, 35) = 3.770 | P=0.0603 |
| Time x Group | F (11, 385) = 0.7320 | P=0.7078 |
| Time x Tail Bleed | F (11, 385) = 1.144 | P=0.3255 |
| Group x Tail Bleed | F (1, 35) = 1.140 | P=0.2929 |
| Time x Group x Tail Bleed | F (11, 385) = 0.7960 | P=0.6441 |
|  |  |  |
| **Infusions** | F (DFn, DFd) | P value |
| Time | F (2.990, 104.6) = 7.295 | P=0.0002 |
| Group | F (1, 35) = 5.997 | P=0.0195 |
| Tail Bleed | F (1, 35) = 0.006830 | P=0.9346 |
| Time x Group | F (11, 385) = 2.414 | P=0.0065 |
| Time x Tail Bleed | F (11, 385) = 1.100 | P=0.3600 |
| Group x Tail Bleed | F (1, 35) = 0.1107 | P=0.7413 |
| Time x Group x Tail Bleed | F (11, 385) = 1.576 | P=0.1035 |
|  |  |  |
| **Intake** | F (DFn, DFd) | P value |
| Time | F (3.136, 109.8) = 6.487 | P=0.0004 |
| Group | F (1, 35) = 6.691 | P=0.0140 |
| Tail Bleed | F (1, 35) = 0.01888 | P=0.8915 |
| Time x Group | F (11, 385) = 2.354 | P=0.0081 |
| Time x Tail Bleed | F (11, 385) = 0.8570 | P=0.5830 |
| Group x Tail Bleed | F (1, 35) = 0.05200 | P=0.8209 |
| Time x Group x Tail Bleed | F (11, 385) = 1.501 | P=0.1283 |

**Table 6.** Results of 3-way ANOVAs on active lever pressing, inactive lever pressing, infusions, and intake during self-administration in male rats.

| **Active Lever** | F (DFn, DFd) | P value |
| --- | --- | --- |
| Time | F (1.512, 57.47) = 15.57 | P<0.0001 |
| Group | F (1, 38) = 0.3422 | P=0.5620 |
| Tail Bleed | F (1, 38) = 0.09390 | P=0.7610 |
| Time x Group | F (11, 418) = 0.5160 | P=0.8927 |
| Time x Tail Bleed | F (11, 418) = 0.2766 | P=0.9899 |
| Group x Tail Bleed | F (1, 38) = 0.5944 | P=0.4455 |
| Time x Group x Tail Bleed | F (11, 418) = 1.146 | P=0.3235 |
|  |  |  |
| **Inactive Lever** | F (DFn, DFd) | P value |
| Time | F (4.064, 154.4) = 3.087 | P=0.0171 |
| Group | F (1, 38) = 0.3801 | P=0.5412 |
| Tail Bleed | F (1, 38) = 0.7072 | P=0.4056 |
| Time x Group | F (11, 418) = 1.367 | P=0.1854 |
| Time x Tail Bleed | F (11, 418) = 1.067 | P=0.3871 |
| Group x Tail Bleed | F (1, 38) = 0.4189 | P=0.5214 |
| Time x Group x Tail Bleed | F (11, 418) = 0.6426 | P=0.7922 |
|  |  |  |
| **Infusions** | F (DFn, DFd) | P value |
| Time | F (3.615, 137.4) = 13.96 | P<0.0001 |
| Group | F (1, 38) = 0.1478 | P=0.7028 |
| Tail Bleed | F (1, 38) = 0.5616 | P=0.4582 |
| Time x Group | F (11, 418) = 0.3664 | P=0.9683 |
| Time x Tail Bleed | F (11, 418) = 0.9060 | P=0.5345 |
| Group x Tail Bleed | F (1, 38) = 0.3584 | P=0.5529 |
| Time x Group x Tail Bleed | F (11, 418) = 0.1714 | P=0.9988 |
|  |  |  |
| **Intake** | F (DFn, DFd) | P value |
| Time | F (3.433, 130.5) = 11.74 | P<0.0001 |
| Group | F (1, 38) = 0.1717 | P=0.6809 |
| Tail Bleed | F (1, 38) = 0.1532 | P=0.6977 |
| Time x Group | F (11, 418) = 0.4391 | P=0.9381 |
| Time x Tail Bleed | F (11, 418) = 0.8326 | P=0.6074 |
| Group x Tail Bleed | F (1, 38) = 0.03821 | P=0.8461 |
| Time x Group x Tail Bleed | F (11, 418) = 0.1471 | P=0.9994 |

**Table 7.** Results of 3-way ANOVAs on active and inactive lever presses during instrumental extinction in female rats.

| **Active Lever** | F (DFn, DFd) | P value |
| --- | --- | --- |
| Time | F (1.934, 67.71) = 36.83 | P<0.0001 |
| Group | F (1, 35) = 0.8970 | P=0.3501 |
| Tail Bleed | F (1, 35) = 4.032 | P=0.0524 |
| Time x Group | F (8, 280) = 1.100 | P=0.3635 |
| Time x Tail Bleed | F (8, 280) = 0.5312 | P=0.8327 |
| Group x Tail Bleed | F (1, 35) = 0.005411 | P=0.9418 |
| Time x Group x Tail Bleed | F (8, 280) = 0.5052 | P=0.8521 |
|  |  |  |
| **Inactive Lever** | F (DFn, DFd) | P value |
| Time | F (3.672, 128.5) = 4.505 | P=0.0026 |
| Group | F (1, 35) = 0.6684 | P=0.4191 |
| Tail Bleed | F (1, 35) = 0.2402 | P=0.6271 |
| Time x Group | F (8, 280) = 1.635 | P=0.1145 |
| Time x Tail Bleed | F (8, 280) = 0.3750 | P=0.9334 |
| Group x Tail Bleed | F (1, 35) = 0.01177 | P=0.9142 |
| Time x Group x Tail Bleed | F (8, 280) = 0.1887 | P=0.9923 |

**Table 8.** Results of 3-way ANOVAs on active and inactive lever presses during reinstatement in female rats.

| **Active Lever** | F (DFn, DFd) | P value |
| --- | --- | --- |
| Time | F (1, 34) = 28.89 | P<0.0001 |
| Group | F (1, 34) = 0.005149 | P=0.9432 |
| Tail Bleed | F (1, 34) = 0.09600 | P=0.7586 |
| Time x Group | F (1, 34) = 0.06118 | P=0.8061 |
| Time x Tail Bleed | F (1, 34) = 0.001394 | P=0.9704 |
| Group x Tail Bleed | F (1, 34) = 0.2257 | P=0.6377 |
| Time x Group x Tail Bleed | F (1, 34) = 0.3799 | P=0.5418 |
|  |  |  |
| **Inactive Lever** | F (DFn, DFd) | P value |
| Time | F (1, 34) = 4.356 | P=0.0444 |
| Group | F (1, 34) = 0.4437 | P=0.5098 |
| Tail Bleed | F (1, 34) = 0.0821 | P=0.7762 |
| Time x Group | F (1, 34) = 0.074 | P=0.7924 |
| Time x Tail Bleed | F (1, 34) = 0.3157 | P=0.5779 |
| Group x Tail Bleed | F (1, 34) = 3.491 | P=0.0703 |
| Time x Group x Tail Bleed | F (1, 34) = 0.6278 | P=0.4337 |

**Table 9.** Results of 3-way ANOVAs on active and inactive lever presses during instrumental extinction in male rats.

| **Active Lever** | F (DFn, DFd) | P value |
| --- | --- | --- |
| Time | F (1.876, 71.29) = 32.20 | P<0.0001 |
| Group | F (1, 38) = 0.0001373 | P=0.9907 |
| Tail Bleed | F (1, 38) = 0.09166 | P=0.7637 |
| Time x Group | F (8, 304) = 0.4361 | P=0.8989 |
| Time x Tail Bleed | F (8, 304) = 1.226 | P=0.2834 |
| Group x Tail Bleed | F (1, 38) = 0.2158 | P=0.6449 |
| Time x Group x Tail Bleed | F (8, 304) = 0.2294 | P=0.9853 |
|  |  |  |
| **Inactive Lever** | F (DFn, DFd) | P value |
| Time | F (3.934, 149.5) = 10.16 | P<0.0001 |
| Group | F (1, 38) = 0.7261 | P=0.3995 |
| Tail Bleed | F (1, 38) = 0.01409 | P=0.9061 |
| Time x Group | F (8, 304) = 1.443 | P=0.1781 |
| Time x Tail Bleed | F (8, 304) = 0.5185 | P=0.8424 |
| Group x Tail Bleed | F (1, 38) = 3.051 | P=0.0888 |
| Time x Group x Tail Bleed | F (8, 304) = 0.7517 | P=0.6457 |

**Table 10.** Results of 3-way ANOVAs on active and inactive lever presses during reinstatement in male rats.

| **Active Lever** | F (DFn, DFd) | P value |
| --- | --- | --- |
| Time | F (1, 36) = 85.96 | P<0.0001 |
| Group | F (1, 36) = 0.5131 | P=0.4784 |
| Tail Bleed | F (1, 36) = 0.2429 | P=0.6251 |
| Time x Group | F (1, 36) = 0.6540 | P=0.4240 |
| Time x Tail Bleed | F (1, 36) = 0.4566 | P=0.5036 |
| Group x Tail Bleed | F (1, 36) = 4.627 | P=0.0383 |
| Time x Group x Tail Bleed | F (1, 36) = 6.740 | P=0.0136 |
|  |  |  |
| **Inactive Lever** | F (DFn, DFd) | P value |
| Time | F (1, 36) = 1.634 | P = 0.2094 |
| Group | F (1, 36) = 2.186 | P = 0.1480 |
| Tail Bleed | F (1, 36) = 0.08959 | P = 0.7664 |
| Time x Group | F (1, 36) = 0.2299 | P = 0.6345 |
| Time x Tail Bleed | F (1, 36) = 0.1357 | P = 0.7147 |
| Group x Tail Bleed | F (1, 36) = 1.688 | P = 0.2021 |
| Time x Group x Tail Bleed | F (1, 36) = 5.221 | P = 0.0283 |

**Table 11.** Results of Student’s t-tests for the elevated plus maze parameters in female rats (Experiment 2).

|  | t (df) | P value |
| --- | --- | --- |
| Time in Open Arms | t (15) = 1.604 | P=0.1296 |
| Time in Closed Arms | t (15) = 0.7210 | P=0.4820 |
| Open Arm Entries | t (15) = 1.054 | P=0.3087 |
| Closed Arm Entries | t (15) = 2.629 | P=0.0182 |
